# Rheumatoid arthritis, psoriatic arthritis, and axial spondyloarthritis epidemiology in England from 2004 to 2020: An observational study using primary care electronic health record data

**DOI:** 10.1016/j.lanepe.2022.100519

**Published:** 2022-10-10

**Authors:** Ian C. Scott, Rebecca Whittle, James Bailey, Helen Twohig, Samantha L. Hider, Christian D Mallen, Sara Muller, Kelvin P. Jordan

**Affiliations:** a Primary Care Centre Versus Arthritis, School of Medicine, Keele University, Keele, UK; b Haywood Academic Rheumatology Centre, Haywood Hospital, Midlands Partnership NHS Foundation Trust, High Lane, Burslem, Staffordshire, UK

**Keywords:** Rheumatoid arthritis, Psoriatic arthritis, Axial spondyloarthritis, Incidence, Prevalence

## Abstract

**Background:**

Contemporary data on rheumatoid arthritis (RA), psoriatic arthritis (PsA) and axial spondyloarthritits (SpA) epidemiology in England are lacking. This knowledge is crucial to planning healthcare services. We updated algorithms defining patients with diagnoses of RA, PsA, and axial SpA in primary care and applied them to describe their incidence and prevalence in the Clinical Practice Research Datalink Aurum, an electronic health record (EHR) database covering ∼20% of England.

**Methods:**

Algorithms for ascertaining patients with RA, axial SpA, and PsA diagnoses validated in primary care EHR databases using Read codes were updated (to account for the English NHS change to SNOMED CT diagnosis coding) and applied. Updated diagnosis and synthetic disease-modifying anti-rheumatic drug code lists were devised by rheumatologists and general practitioners. Annual incidence/point-prevalence of RA, PsA, and axial SpA diagnoses were calculated from 2004 to 2020 and stratified by age/sex.

**Findings:**

Point-prevalence of RA/PsA diagnoses increased annually, peaking in 2019 (RA 0·779% [95% confidence interval (CI) 0·773, 0·784]; PsA 0·287% [95% CI 0·284, 0·291]) then falling slightly. Point-prevalence of axial SpA diagnoses increased annually (except in 2018/2019), peaking in 2020 (0·113% [95% CI 0·111, 0·115]). RA diagnosis annual incidence was higher between 2013-2019 (after inclusion in the Quality and Outcomes Framework, range 49·1 [95% CI 47·7, 50·5] to 52·1 [95% CI 50·6, 53·6]/100,000 person-years) than 2004-2012 (range 34·5 [95% CI 33·2, 35·7] to 40·0 [95% CI 38·6, 41·4]/100,000 person-years). Increases in the annual incidence of PsA/axial SpA diagnosis occurred following new classification criteria publication. Annual incidence of RA, PsA and axial SpA diagnoses fell by 40·1%, 67·4%, and 38·1%, respectively between 2019 and 2020, likely reflecting the COVID-19 pandemic's impact on their diagnosis.

**Interpretation:**

Recorded RA, PsA, and axial SpA diagnoses are increasingly prevalent in England, underlining the importance of organising healthcare services to provide timely, treat-to-target care to optimise the health of >1% of adults in England.

**Funding:**

National Institute for Health and Care Research (NIHR300826).

**Research in context**

**Evidence before this study**

To plan healthcare provision for people with rheumatoid arthritis (RA), psoriatic arthritis (PsA), and axial spondyloarthritis (SpA) in European countries, it is crucial to know the proportion of their populations diagnosed with these conditions, which age groups are most affected, and how this is changing over time.

We conducted a Pubmed search (until December 2021) to identify systematic literature reviews examining the global incidence and/or prevalence of RA, PsA, and axial SpA. We also searched Pubmed for observational studies examining this in the Clinical Practice Research Datalink and The Health Improvement Network, which represent the main primary care electronic health record databases with national coverage available in England.

Four recently published systematic reviews were identified (published in 2018 for PsA and axial SpA, and 2016 and 2020 in RA). These showed considerable heterogeneity in the reported incidence and prevalence of these conditions across studies, which varied by study geography and methodology. Most research focused on RA; there was very limited published data on axial SpA. The systematic reviews demonstrated uncertainty in whether the incidence and prevalence of these conditions has changed over time.

One study examined trends in the incidence and prevalence of RA over time in the Clinical Practice Research Datalink GOLD. This reported that the prevalence of patients diagnosed with RA increased from 1990 to 2005, then fell and plateaued between 2007 and 2014. In contrast, incidence rates were high in the early 1990s, falling rapidly, increasing, and then falling and plateauing between 2007 and 2014. Its use of single Read codes to identify patients with RA and inclusion of historical data from the 1990s (when coding practices most likely differed and electronic health record use was less widespread) may explain some of these findings.

**Added value of this study**

This study has updated validated algorithms to ascertain patients with diagnoses of the three main forms of inflammatory arthritis in primary care electronic health record databases and applied them in the Clinical Practice Research Datalink Aurum (a primary care electronic health record dataset covering approximately 20% of the English population) to generate contemporary data on their annual incidence and point prevalence.

It demonstrates that the prevalence of these three diagnoses has increased by at least 40% between 2004 and 2020. In 2020 >1% of all adults and >2·5% of those aged >65 years had a diagnosis of RA, PsA or axial SpA. Several factors influenced the incidence of coded diagnoses, with the annual incidence of RA diagnoses increasing by at least a quarter after its introduction into the Quality and Outcomes Framework (which reimburses practices for undertaking specific activities in patients with RA), and the incidence of all three conditions falling by ≥38% between 2019 and 2020 (notable for the onset of the COVID-19 pandemic). The introduction of new classification criteria for axial SpA was followed by an increase in its incidence, particularly in women, likely reflecting the change in disease definition to incorporate magnetic resonance imaging (in addition to plain radiograph) identified sacroiliitis. Overall, the updated algorithms we used to ascertain patients with diagnoses of RA, PsA, and axial SpA led to incidence and prevalence estimates broadly consistent with published studies, supporting their use in future research.

**Implications of all the available evidence**

The increasing prevalence of patients diagnosed with RA, PsA, and axial SpA in English primary care between 2004 and 2020 highlights a need to ensure that NHS services are adapted accordingly. Their high prevalence amongst people aged over 65 years underlines the importance of considering older people in service provision.

Alt-text: Unlabelled box

## Introduction

Inflammatory arthritis groups together conditions causing autoimmune-driven joint inflammation. It has three main forms, comprising rheumatoid arthritis (RA), psoriatic arthritis (PsA), and axial spondyloarthritis (SpA). These are treated in a similar manner using immunosuppressive treatments, cause long-term pain and disability,^1^, ^2^, ^3^ and incur substantial personal and societal costs, with an estimated annual cost to the UK economy from sick leave and work-related disability for people with RA alone totalling £1·8 billion.^4^ The evidence that early immunosuppressive therapy optimises the outcomes of patients with inflammatory arthritis is considerable. Ensuring that healthcare services are optimised to deliver prompt and appropriate care to patients with RA, PsA, and axial SpA is therefore crucial. Having a detailed understanding of the proportion of the population that are diagnosed with these conditions, and how this varies by age groups and over time is key to informing healthcare delivery.

Several recent systematic literature reviews have examined the global incidence and prevalence of these three conditions.^5^, ^6^, ^7^, ^8^ They demonstrate significant heterogeneity across studies in incidence and prevalence estimates, which vary substantially across geographical regions and by study methodology. The pooled prevalence estimates for RA and PsA were 0·46% (67 studies; I^2^=99·9%)^5^ and 0·13% (28 studies; I^2^=99·3%),^7^ respectively. Three studies were identified in the systematic review of axial SpA epidemiology; all estimated prevalence by screening people to establish if they met classification criteria, with prevalence estimates ranging from 0·13 to 1·4%.^8^ Identified cohort studies examining RA incidence provided conflicting results; some suggested a decline and others an increase over time.^6^ Only nine studies were identified that examined PsA incidence; the pooled incidence estimate was 8·26 per 100,000.^7^ No studies of axial SpA incidence were identified.^8^

Little data exist on the incidence and prevalence of RA, PsA, and axial SpA and their trends in England. The fragmented nature of English secondary care digital systems - with NHS trusts using a variety of outdated “legacy” systems that are unable to interact with each other - means that primary care electronic health record (EHR) databases are best placed to understand inflammatory arthritis epidemiology. To date, two studies have examined RA and PsA epidemiology in two of the most used UK primary care EHR databases for research, The Health Improvement Network (THIN), and Clinical Practice Research Datalink (CPRD) GOLD. Within CPRD GOLD, an analysis of the incidence and prevalence of patients receiving a Read code suggestive of an RA diagnosis reported that the prevalence increased from 1990 to 2005, then fell and plateaued between 2007 and 2014.^9^ Incidence rates were high in the early 1990s, falling rapidly from 74.6 to 35.8 per 100,000, increasing to 52.0 per 100,000, and then falling and plateauing between 2007 and 2014. Its use of single Read codes to identify patients with RA and inclusion of historical data from the 1990s (when data quality and coding practices likely differed to more recent time-periods) may explain some of these findings. Within THIN, 0·19% of the population (aged between 18 and 90 years) had received at least one Read code for PsA at some point between 1994 and 2010^10^; the time-trends in prevalence were not examined. To our knowledge, no national data on the incidence and prevalence of patients diagnosed with axial SpA in England exist.

To use primary care EHRs for epidemiological inflammatory arthritis research, it is first crucial to develop methods that reliably define patients with these diagnoses. As primary care inflammatory arthritis diagnosis codes do not always equate with having these conditions^11^ studies in GOLD and THIN used algorithms, combining serial diagnosis codes with/without specialist prescriptions.^12^^,^^13^ Since their development, NHS coding has changed from Read^14^ to SNOMED CT codes,^15^ expanding the number of available diagnosis codes. The aim of this study was, therefore, to update previously validated algorithms defining patients with diagnoses of RA, PsA, and axial SpA in primary care EHRs^12^^,^^13^ accounting for the change in NHS coding processes and apply these in CPRD Aurum – a large primary care EHR database currently covering approximately 20% of the English population^16^ – to generate contemporary data on the incidence and prevalence of patients with diagnoses of these conditions.

## Methods

### EHR database

Aurum contains routinely collected data from GP practices using EMIS Web EHR software. It currently includes data from 1,491 practices, 99% of which are in England.^16^

### Diagnosis codes

89, 6, and 5 Read/SNOMED codes for diagnoses of RA, PsA, and axial SpA, respectively were generated through consensus methods involving consultant rheumatologists (ICS/SH) and GPs (CDM/HT) (details in Supplementary Table 1; code lists available in Supplementary Tables 2 to 4 and online at https://www.keele.ac.uk/mrr/codelists/otherdefinitions/). Read Codes are a coded thesaurus of clinical terms used in the NHS since 1985 to code diagnoses, patient findings, and procedures in their EHRs.^14^ SNOMED CT is an alternative vocabulary for recording diagnoses and other clinical information in EHRs.^15^ Since April 2020 all NHS services in England have moved from Read to SNOMED CT codes.

### Diagnosis algorithms

#### RA

The algorithm developed in the General Practice Research Database (CPRD's predecessor) was updated to include SNOMED codes. The original algorithm has a sensitivity of 84% and specificity of 86% for an RA diagnosis (in patients with ≥one RA Read code).^11^ Prior to algorithm application Read codes are grouped based on RA strength of evidence. Group 1 (“strong” evidence) comprises seropositive/erosive RA codes. Group 2 (“fairly strong” evidence) comprises “RA” codes e.g., RA of knee. Group 3 (“fairly weak” evidence) comprises codes for RA systemic manifestations e.g., Felty's syndrome. Group 4 (“weak” evidence) comprises other codes. The algorithm classifies patients as having RA if they meet one of two criteria: (1) have ≥one RA Read code and ≥one disease-modifying anti-rheumatic drug (DMARD) prescription after the first RA code with no alternative DMARD indication (no Read code for an alternative indication for 5 years pre-first DMARD prescription); (2) have: (a) ≥two RA Read codes (on different dates); (b) no alternative diagnosis (alternative inflammatory arthritis type) after the final code; and (c) a code from groups 1/2, as opposed to 3/4.

To update and apply the algorithm, accounting for the move to SNOMED coding, ICS, SH, CDM, and HT allocated Read/SNOMED codes to evidence groups, devised synthetic DMARD product code lists, and identified Read/SNOMED codes for alternative DMARD indications/alternative diagnoses superseding RA (Supplementary Tables 5 and 6; code lists publicly available online at https://www.keele.ac.uk/mrr/codelists/otherdefinitions/). Biologic/targeted synthetic DMARDs were not considered (secondary care prescribed, and in England require previous synthetic DMARD prescriptions).

#### PsA

As the positive predictive value (PPV) of a single PsA Read code for a GP-confirmed PsA diagnosis is high (85% in THIN),^17^ we considered the presence of a single PsA Read/SNOMED code to be evidence for a diagnosis.

#### Axial SpA

Dubreuil et al concluded that two Read codes for ankylosing spondylitis (AS) ≥seven days apart optimised the PPV (88·6%) for identifying patients with a GP-confirmed AS diagnosis in THIN.^13^ We applied this in Aurum and also included non-radiographic axial SpA Read/SNOMED codes.

### Study sample

We included patients ever-receiving a Read/SNOMED code for RA, PsA, or axial SpA that were: (a) aged ≥18 years at index date (date of first inflammatory arthritis code), and (b) contributed data to English GP practices in Aurum at any time-point between 01/01/2004 and 31/12/2020.

### Statistical analysis

#### Proportion meeting diagnosis algorithms

The proportion of patients ever-receiving at least one Read/SNOMED code for RA and axial SpA and currently contributing data who fulfilled the algorithms was determined annually.

#### Demographics

In patients ever-meeting the RA algorithm, their age (at end of calendar year) and sex were summarised in each calendar-year they contributed data to using means/proportions. This was repeated for PsA and axial SpA.

#### Incidence and prevalence

Annual incidence of RA diagnosis was calculated per 100,000 person-years at risk (with 95% confidence intervals [CI]). The denominator was all adults (aged ≥18 years) registered during the calendar-year specified who were never coded as having RA before the 1^st^ of January of that calendar-year, or in those patients first registered in the preceding calendar-year, within 365 days of registration date. The numerator was patients meeting the RA diagnosis algorithm during that calendar-year. The point-prevalence of RA diagnosis in each calendar-year was calculated with 95% CIs. In each calendar-year, the denominator was all adults (aged ≥18 years) registered on the 31st December of that calendar-year. The numerator was all patients meeting the RA diagnosis algorithm prior to/on the 31st December of that calendar-year. Annual incidence and point-prevalence was further reported stratified by age and sex, and further reported stratified by age in males and females separately (merging age groups containing <5 individuals in line with CPRD requirements). This was repeated for PsA and axial SpA.

To ensure temporal changes in incidence/prevalence were not due to confounding arising from changes in English population age/sex structure, we calculated age- and sex-standardised diagnosis incidence and prevalence for each calendar-year, using the 2020 English population structure as a reference.^18^

#### Frequency of diagnosis codes used in incident diagnoses

Frequency counts for each initial Read/SNOMED code used in patients with an incident diagnosis of RA, PsA, and axial SpA in each calendar year were calculated.

### Statistical programmes

Data management and analysis was conducted in R/Stata/MP version 17.0.

### Patient and public involvement

We are co-designing dissemination messages with a patient advisory group comprising four public contributors with inflammatory arthritis.

### Ethics

CPRD has ethics approval from the Health Research Authority to support research using anonymised patient data. As patients contributing data to CPRD cannot be identified from the data made available to researchers, individual patient consent is not required. The study was approved by the CPRD Research Data Governance Process (ref 20_000244; protocol made available to this manuscript's reviewers).

### Role of funding source

The funders played no role in the writing of the manuscript or the decision to submit it for publication.

## Results

### Number of diagnosis codes and proportion meeting diagnosis algorithms

#### RA

**Figure 1:**
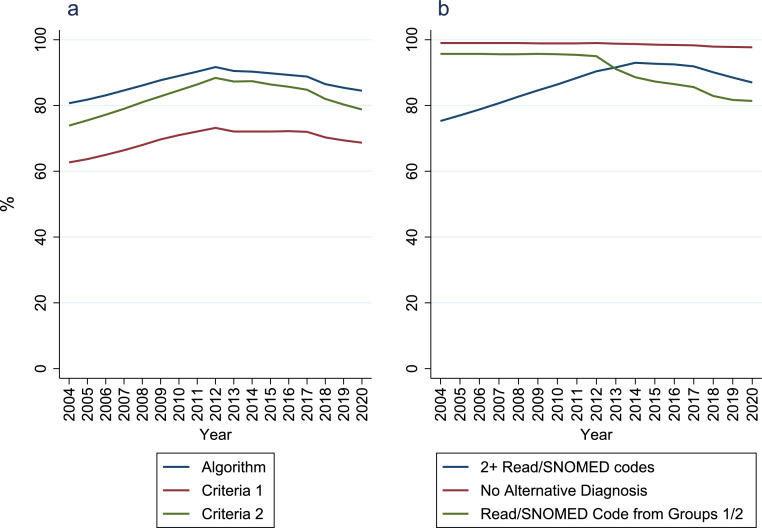
**Percentage of patients with at least 1 Read/SNOMED code for RA who meet the diagnosis algorithm**. Panel A = percentage of patients ever receiving an RA Read/SNOMED code that meet the algorithm and each of its criteria in each calendar-year; panel B = percentage of patients ever receiving an RA Read/SNOMED code that meet each criteria 2 component in each calendar-year; 2+ Read/SNOMED codes = having ≥2 Read/SNOMED codes (on different dates); no alternative diagnosis = no Read/SNOMED code for an alternative form of IA after the final RA Read/SNOMED code; Read/SNOMED code from groups 1/2 = having a Read/SNOMED code from strength of evidence code groups 1 (“strong” evidence) or 2 (“fairly strong” evidence) as opposed to 3 (“fairly weak” evidence) or 4 (“weak” evidence).

The number of patients ever receiving an RA Read/SNOMED code and contributing data in that calendar year increased from 63,781 in 2004 to 107,168 in 2020. The proportion of patients ever receiving an RA Read/SNOMED code fulfilling the diagnosis algorithm increased from 80·7% in 2004 to 91·7% in 2012, before declining to 84·5% in 2020 (Figure 1; Supplementary Table 7). Over time the proportion receiving ≥two Read/SNOMED codes increased and the proportion with codes from evidence groups 1/2 decreased.

#### PsA

The number of patients ever receiving a PsA Read/SNOMED code and contributing data in that calendar year increased from 13,594 in 2004 to 32,770 in 2020 (Supplementary Table 8).

#### Axial SpA

The number of patients ever receiving an axial SpA Read/SNOMED code and contributing data in that calendar year increased from 13,210 in 2004 to 21,343 in 2020 (Supplementary Table 9). The proportion of patients ever receiving a Read/SNOMED code for axial SpA fulfilling the diagnosis algorithm increased from 56·5% in 2004 to 60·6% in 2020.

### Patient demographics

Most patients meeting the RA diagnosis algorithm were female (70·5% to 71·2% across years; Supplementary Table 10). The sex distribution for those diagnosed with PsA was more equal, but the proportion of females increased over time (47·8% in 2004; 51·1% in 2020; Supplementary Table 11). Most patients meeting the axial SpA diagnosis algorithm were male, but the proportion of females increased over time (21·4% in 2004; 29·0% in 2020; Supplementary Table 12). Mean ages for patients with RA diagnoses were in the 7^th^ decade and PsA/axial SpA the 6^th^ decade.

### Incidence

#### RA

**Figure 2:**
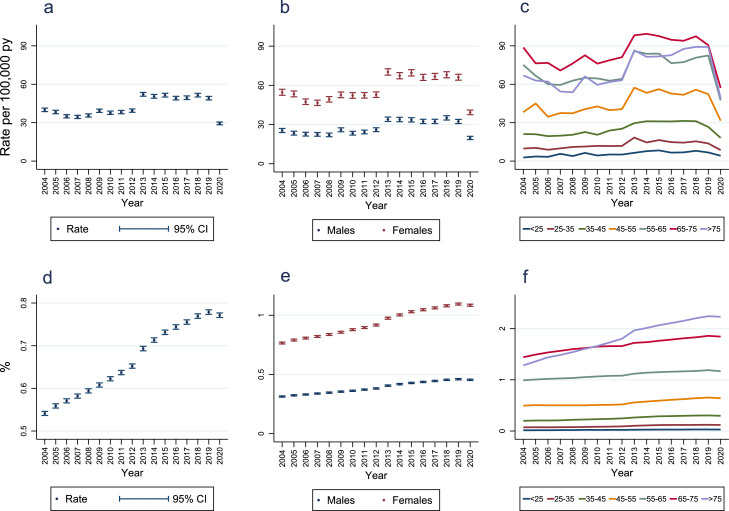
**Annual incidence and point-prevalence of rheumatoid arthritis diagnoses**. Panel A = annual incidence; Panel B = incidence stratified by sex (<5 patients had indeterminate sex recorded and were excluded from this analysis); Panel C = incidence stratified by age-bands (<25: < 25 years; 25-35: ≥25 to <35 years; 35-45: ≥35 to <45 years; 45-55: ≥45 to <55 years; 55-65: ≥55 to <65 years; 65-75: ≥65 to <75 years; >75: ≥75 years); Panel D = overall prevalence; Panel E = prevalence stratified by sex (<5 patients had indeterminate sex recorded and were excluded from this analysis); Panel F = prevalence stratified by age-bands (<25: < 25 years; 25-35: ≥25 to <35 years; 35-45: ≥35 to <45 years; 45-55: ≥45 to <55 years; 55–65: ≥55 to <65 years; 65-75: ≥65 to <75 years; >75: ≥75 years); py = person-years; CI = confidence interval.

Annual incidence of RA diagnosis was relatively stable from 2004-2012, ranging from 34·5 (95% CI 33·2, 35·7) to 40·0 (95% CI 38·6, 41·4) per 100,000 person-years (Figure 2; Supplementary Table 13). Subsequently, from 2013-2019 it was higher and relatively stable, ranging from 49·1 (95% CI 47·7, 50·6) to 52·1 (95% CI 50·6, 53·6) per 100,000 person-years, before falling in 2020 to 29·4 (95% CI 28·3, 30·5) per 100,000 person-years. These time-periods are notable for RA's inclusion in the Quality and Outcomes Framework (QOF) in 2013,^19^ and England's COVID-19 pandemic (onset March 2020).

Annual incidence of RA diagnosis in females was approximately twice that in males (Figure 2; Supplementary Table 13). Highest incidence rates in each calendar-year were seen in the 65-75-year age group (Figure 2; Supplementary Table 14). Similar incidence time-trends were seen in each age group, excepting a larger decrease in incidence between 2019 and 2020 in older age groups. Similar patterns of annual incidence rates in males and females stratified by age groups were seen, except that in males incidence rates in the ≥75-year age group were higher than in the ≥55 to <65-year age group with the opposite observed in females (Supplementary Figure 1).

#### PsA

**Figure 3:**
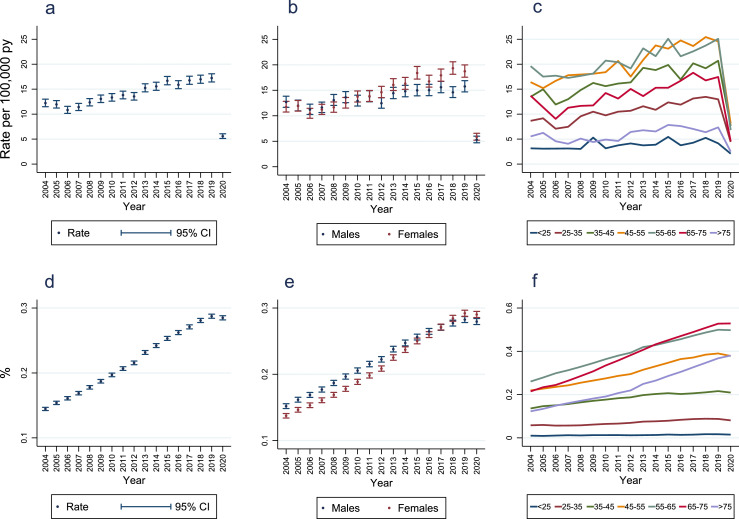
**Annual incidence and point-prevalence of psoriatic arthritis diagnoses**. Panel A = incidence; Panel B = incidence stratified by sex (<5 patients had indeterminate sex recorded and were excluded from this analysis); Panel C = incidence stratified by age-bands (<25: < 25 years; 25-35: ≥25 to <35 years; 35-45: ≥35 to <45 years; 45-55: ≥45 to <55 years; 55-65: ≥55 to <65 years; 65-75: ≥65 to <75 years; >75: ≥75 years); Panel D = overall prevalence; Panel E = prevalence stratified by sex (<5 patients had indeterminate sex recorded and were excluded from this analysis); Panel F = prevalence stratified by age-bands (<25: < 25 years; 25-35: ≥25 to <35 years; 35-45: ≥35 to <45 years; 45-55: ≥45 to <55 years; 55-65: ≥55 to <65 years; 65-75: ≥65 to <75 years; >75: ≥75 years); py = person-years; CI = confidence interval.

Annual incidence of PsA diagnosis fell slightly from 12·2 (95% CI 11·5, 13·0) to 10·8 (95% CI 10·1, 11·6) per 100,000 person-years between 2004 and 2006 (Figure 3; Supplementary Table 15). A persistent upward trend from 2006 (the year that the new ClASsification criteria for Psoriatic ARthritis [CASPAR] were introduced)^20^ occurred, peaking at 17·2 (95% CI 16·4, 18·1) per 100,000 person-years in 2019, before falling to 5·6 (95% CI 5·1, 6·1) per 100,000 person-years in 2020.

Changes in the female:male ratio of the annual incidence of PsA diagnosis occurred over time (Figure 3, Supplementary Table 15). From 2004-2011 incidence rates were similar in both sexes. From 2012 PsA diagnosis incidence was 1·1 to 1·3 times higher in females; the largest difference occurred in 2018 (incidence 19·3 and 14·6 per 100,000 person-years in females and males, respectively). In all calendar-years, annual PsA diagnosis incidence was highest in the ≥45 to <55 and ≥55 to <65 age groups (Figure 3; Supplementary Table 16). Similar time-trends were observed in each age group, excepting larger decreases in annual incidence between 2019 and 2020 in age groups with the highest 2019 incidence. Similar patterns and trends in incidence rates in males and females stratified by age groups were seen, except incidence rates in many age groups increased at a greater rate between 2006 and 2020 in females than males (Supplementary Figure 1).

#### Axial SpA

**Figure 4:**
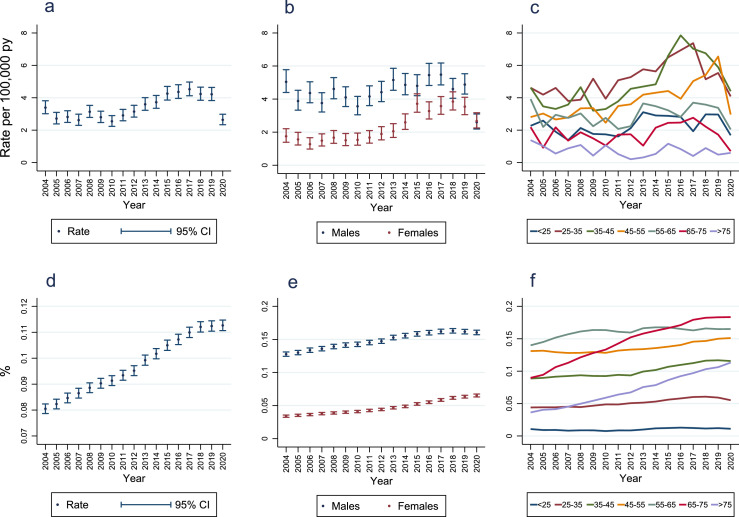
**Annual incidence and point-prevalence of axial spondyloarthritis diagnoses**. Panel A = incidence; Panel B = incidence stratified by sex; Panel C = incidence stratified by age-bands (<25: < 25 years; 25-35: ≥25 to <35 years; 35-45: ≥35 to <45 years; 45-55: ≥45 to <55 years; 55-65: ≥55 to <65 years; 65-75: ≥65 to <75 years; >75: ≥75 years); Panel D = overall prevalence; Panel E = prevalence stratified by sex; Panel F = prevalence stratified by age-bands (<25: < 25 years; 25-35: ≥25 to <35 years; 35-45: ≥35 to <45 years; 45-55: ≥45 to <55 years; 55-65: ≥55 to <65 years; 65-75: ≥65 to <75 years; >75: ≥75 years); py = person-years; CI = confidence interval.

Annual axial SpA diagnosis incidence fell slightly from 3·4 (95% CI 3·0, 3·8) to 2·7 (95% CI 2·4, 3·1) per 100,000 person-years from 2004 to 2005 (Figure 4; Supplementary Table 17). It was then relatively stable, before having a persistent upward trend between 2010 (year after Assessment of SpondyloArthritis international Society [ASAS] classification criteria publication)^21^ and 2015, followed by relative stability until declining between 2019 and 2020 from 4·2 (95% CI 3·8, 4·6) to 2·6 (95% CI 2·3, 3·0) per 100,000 person-years.

Between 2014 and 2019 the annual incidence of axial SpA diagnosis in males was relatively stable, before falling in 2020 (Figure 4; Supplementary Table 17). In contrast, the trends in annual incidence for females mirrored overall trends, increasing from 2010 to 2015, followed by relative stability until declining in 2020. In all years, annual axial SpA diagnosis incidence was higher in males except 2020 when similar rates were seen in both sexes. In all but one calendar-year annual incidence rates were highest in the ≥25 to <35 or ≥35 to <45 age groups (Figure 4; Supplementary Table 18). In both males and females, incidence rates were lower in those aged ≥55 years than those aged <35 years and ≥35 to <55 years (Supplementary Figure 1). In females, following ASAS criteria publication incidence rates increased most in females aged ≥35 to <55 years.

### Point-prevalence

#### RA

From 2004-2019 RA diagnosis point-prevalence increased annually from 0·541% (95% CI 0·536, 0·546) to 0·779% (95% CI 0·774, 0·784) (Figure 2; Supplementary Table 19). Between 2004 and 2020 the prevalence increased by 42·5%.

Point-prevalence of RA diagnosis was 2·38 to 2·45 times higher in women than men across calendar-years, increasing over time in all age categories (Figure 2; Supplementary Tables 19 and 20). In 2004, the highest prevalence was in those aged ≥65 to <75 years; over time this changed to being highest in those aged ≥75 years. In 2020, 1·845% (95% CI 1·820, 1·870) and 2·231% (95% CI 2·202, 2·260) of those aged ≥65 to <75 years and ≥75 years had a diagnosis of RA, respectively (Supplementary Table 20). Similar patterns of prevalence in males and females stratified by age groups were observed over time (Supplementary Figure 2).

#### PsA

From 2004–2019 point-prevalence of PsA diagnosis increased annually from 0·145% (95% CI 0·142, 0·147) to 0·287% (95% CI 0·284, 0·291) (Figure 3; Supplementary Table 21). Between 2004 and 2020 the prevalence increased by 96·6%.

Minor sex differences in PsA diagnosis point-prevalence were seen, which changed over time (Figure 3; Supplementary Table 21): in 2004–2016 PsA diagnosis point-prevalence was 1·02 to 1·11 times higher in males than females; in 2017 the sex distribution was equal; in subsequent years point-prevalence was 1·03 to 1·04 times higher in females than males. Patterns in the point-prevalence of PsA diagnosis by age-groups changed over time being highest in those aged ≥55 to <65 until 2014 when it became highest in those aged ≥65 to <75 (Figure 3; Supplementary Table 22). In 2020, 0·529% (95% CI 0·516, 0·542) of those aged ≥65 to <75 years had a diagnosis of PsA (Supplementary Table 22). Similar patterns of prevalence in males and females stratified by age groups were observed over time (Supplementary Figure 2).

#### Axial SpA

From 2004-2020 axial SpA diagnosis point-prevalence increased annually from 0·081% (95% CI 0·079, 0·082) to 0·113% (95% CI 0·111, 0·115) (Figure 4; Supplementary Table 23), representing a 39·5% increase in prevalence over 17 years.

Axial SpA diagnoses were more prevalent in men than women. The male:female ratio fell over time from 3·76 in 2004 to 2·46 in 2020 (Figure 4; Supplementary Table 23). In all age groups except <25 years, axial SpA diagnosis point-prevalence increased over time (Figure 4; Supplementary Table 24), with the greatest increases seen in the age groups ≥65 to <75 and ≥75. In 2020, 0·183% (95% CI 0·176, 0·191) and 0·113% (95% CI 0·107, 0·12) of those aged ≥65 to <75 years and ≥75 years had a diagnosis of axial, respectively (Supplementary Table 24). Between 2004 and 2020 the point-prevalence of axial SpA diagnoses increased proportionally more from their baseline values in females than males in all age groups, particularly those aged ≥25 to <35 and ≥35 to <45 years (Supplementary Figure 2).

### Age and sex standardised incidence/prevalence

**Figure 5:**
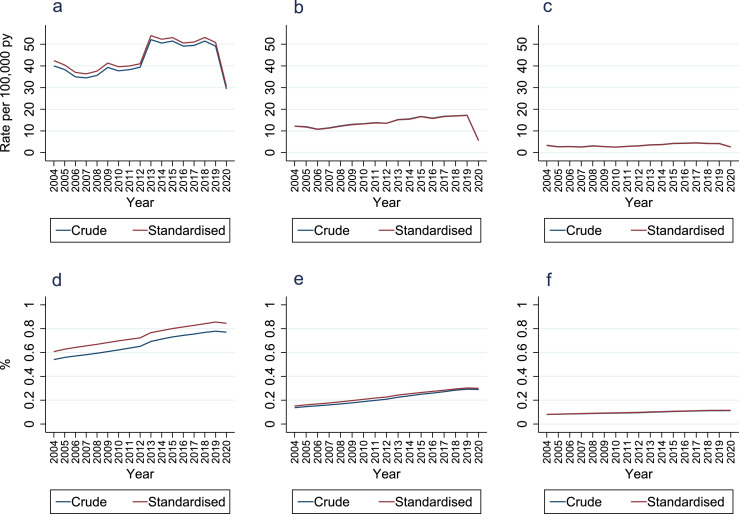
**Annual crude versus age and sex standardised incidence and point-prevalence of inflammatory arthritis diagnoses**. Panel A = rheumatoid arthritis incidence; Panel B = psoriatic arthritis incidence; Panel C = axial spondyloarthritis incidence; Panel D = rheumatoid arthritis prevalence; Panel E = psoriatic arthritis prevalence; Panel F = axial spondyloarthritis prevalence.

Standardising annual incidence and point-prevalence by age and sex led to slightly higher annual incidence and point-prevalence for RA diagnoses, and slightly lower annual incidence and higher point-prevalence for PsA and axial SpA diagnoses but did not alter time-trends (Figure 5; Supplementary Tables 13, 15, 17, 19, 21, and 23).

### Frequency of diagnosis codes used in incident diagnoses

In all calendar years the commonest initial code used in patients with an incident RA diagnosis mapped to the term “rheumatoid arthritis” (accounting for 34.0% to 78.8% of codes; Supplementary Table 25). In patients with an incident PsA diagnosis three codes accounted for most used; all mapped to one SNOMED concept ID (preferred term of “psoriasis with arthropathy”). In 2020, these accounted for 98.8% of initial codes. In people with an incident axial SpA diagnosis, most (>98%) initial codes from 2004 to 2013 were for “ankylosing spondylitis”. From 2014 this declined, with codes for “axial spondyloarthritis” increasingly used (accounting for 31.5% of codes in 2020). Codes for “non-radiographic axial spondyloarthritis” were rare but increased over time (accounting for 2.9% of codes in 2020).

## Discussion

This national study, which utilised EHR data from over 1,400 general practices spanning 20% of England and updated validated algorithms defining patients diagnosed with RA, PsA, and axial SpA, has demonstrated an increased annual prevalence of diagnosed inflammatory arthritis, which rose by 42·5%, 96·6%, and 39·5% for RA, PsA, and axial SpA, respectively, between 2004 and 2020. Whilst this has probably arisen, at least in part, from improvements in recognising patients with inflammatory arthritis and better coding practices, the fact that the prevalence has increased most in older age groups suggest that a key driver for the increasing prevalence we observed is that many people diagnosed with these conditions live with them for many years. This has important implications for health services, with rheumatology departments accounting for approximately 9% of the average NHS trust's total medication 2019/2020 spend (due to the widespread prescribing of high-cost biologic drugs to treat inflammatory arthritis)^22^ and National Institute for Health and Care Excellence (NICE) advocating that patients with RA receive advice within 1-working-day of contacting rheumatology services for disease flares/treatment side-effects, alongside comprehensive rheumatology-coordinated annual reviews. Furthermore, the fact that one of these conditions is now a recognised diagnosis in >2·5% of people aged ≥65 years highlights the importance of considering older people in planning inflammatory arthritis healthcare services, and ensuring that the rapid move to digital healthcare does not detrimentally affect them (with older people less likely to have internet access,^23^ use the internet,^24^ and possess the essential digital skills to access it independently).^25^

We identified three national developments corresponding to time-periods when trends in the annual incidence of new diagnoses changed for the various forms of inflammatory arthritis. First, is RA's 2013 introduction into the QOF component of the GP contract, which aligned with a sustained incidence increase. QOF reimburses practices for specific activities, which for RA included maintaining a register and evaluating cardiovascular/fracture risks.^19^ It is recognised that including conditions in QOF affects their diagnosis coding (making it more specific)^26^ and prevalence estimates.^27^ Our finding may reflect increased efforts by primary care to enter RA diagnosis codes on receiving rheumatologist's letters detailing incident cases, alongside actively searching for patients with unrecorded prevalent RA. Whilst highly-probable this increase is artefactual, the result – better primary care RA diagnosis recording – is likely to lead to better patient outcomes. Second, is the introduction of new PsA and axial SpA classification criteria, which aligned with sustained increases in their annual incidence of new diagnoses. Whilst classification criteria are not diagnostic criteria, in clinical practice physicians often use such criteria to support diagnostic decisions, and both criteria provide a broader concept of what these conditions represent (with ASAS criteria incorporating MRI-demonstrated sacroiliitis, and CASPAR criteria extending the presence of psoriasis to family members). Third, is the COVID-19 pandemic onset, following which a marked reduction in the incidence of all evaluated forms of inflammatory arthritis occurred. Many studies have focused on the pandemic's direct impacts on patients with inflammatory rheumatic diseases (IRDs), demonstrating an increased risk of COVID-19 infection compared to the general population,^28^ but few have considered its indirect impacts. Two surveys suggest this is substantial, with 58% of rheumatology healthcare professionals perceiving a longer interval between IRD symptom onset and initial consultation during the pandemic,^29^ and a 13·6% reduction in adults with rheumatic diseases being in full-time employment during the pandemic. Our study provides indirect evidence of the pandemic's impact on the national care of people with RA, PsA, and axial SpA, with the marked decline in the annual incidence of new diagnoses at the onset of the pandemic suggesting many cases may have gone undiagnosed during 2020.

In contrast to classification criteria for PsA and axial SpA, we did not observe any clear relationship between the introduction of the 2010 EULAR/ACR classification criteria for RA and changes in the annual incidence of RA diagnoses.^30^ Whilst the annual incidence of RA diagnoses was slightly higher in 2011 and 2012 compared to 2010, the increase was minimal; it then markedly increased in 2013 when RA was included in QOF. Our findings in 2011 and 2012 suggest that the updated RA classification criteria did not substantially impact on the rates of incident RA diagnoses, replicating previous research in CPRD GOLD.^9^ One potential explanation for these findings is that clinicians were already applying the classification criteria's principles (early diagnosis through considering acute phase response markers, anti-CCP status, and small joint involvement). Alternatively, it may be that they did impact on incidence rates, but this was delayed by several years until 2013, by which point their effect occurred concurrently with the impact of QOF.

We observed a change in the sex distributions of people with diagnoses of PsA and axial SpA over time. The greater increase in the annual incidence of PsA diagnosis in women than men replicates North American^31^ and Taiwanese^32^ studies but is not easily explainable. The increase in annual incidence of axial SpA diagnosis in women but not men beginning the year after ASAS classification criteria publication (introducing the concept of non-radiographic axial SpA) is consistent with other studies indicating that whilst AS is male-predominant, non-radiographic axial SpA is commoner in women.^33^ Our finding of a gradual increase in the frequency of codes for “axial spondyloarthritis” amongst people with incident axial SpA diagnoses (with a corresponding decline in the frequency of codes for “ankylosing spondylitis”) indicate that this broader disease definition is being used in primary care.

We did not include patients with diagnosis codes for both psoriasis and axial SpA in our definition of having a diagnosis of PsA. The issue of how to classify axial involvement in patients with psoriasis is an area of ongoing debate, with uncertainty existing as to whether patients with psoriasis and inflammatory axial disease should be diagnosed as having “PsA with axial involvement” or “axial SpA with psoriasis”. This issue is currently the subject of an international collaborative study (the Axial Involvement in Psoriatic Arthritis [AXIS] study) whose aim is to develop classification criteria and a unified nomenclature for axial involvement in PsA.^34^ However, as primary care coding practices now generally focus on the inputting of inflammatory arthritis codes on receipt of a rheumatology department letter confirming their presence, it would be expected that the coded diagnoses in Aurum would largely match those made on rheumatologists’ letters, with patients assigned a diagnosis of PsA or axial SpA accordingly.

The most recent data on English RA incidence/prevalence is from CPRD GOLD, with an incidence of patients with a Read code suggestive of an RA diagnosis of 38·1/100,000 person-years and point-prevalence of 0·67% reported in 2014.^9^ In our study, the incidence of RA diagnoses in 2014 was 50·5/100,000 person-years and point-prevalence 0·713%. Whilst marginally higher, it is well recognised that RA incidence and prevalence estimates vary by methodology,^5^^,^^6^ our results are within the range reported,^5^^,^^6^ and have expected age and sex distributions (being commoner with increasing age and in females). On a global level, PsA prevalence is well-described, with a systematic literature review and meta-analysis reporting a pooled prevalence of 0·133% (range 0·02% to 0·67%).^7^; In England, in the THIN database, 0·19% of the population (aged between 18 and 90 years) had received at least one Read code for PsA at some point.^10^ In our study, PsA diagnosis annual point-prevalence ranged from 0·145% to 0·287%. Limited data on English PsA incidence exist. In other countries, PsA incidence ranges from 3·02 to 41·30/100,000^7^; our estimated annual incidence in English primary care was 10·8 to 17·2/100,000 person-years. Comparing our axial SpA results to published studies is challenging, as most evaluated AS^8^^,^^35^ and those considering the broader concept of axial SpA (including patients with both AS and non-radiographic axial SpA) often estimated prevalence by screening people.^36^^,^^37^ These reported an overall AS prevalence of 0·18%^38^ and incidence of 0·44 to 15/100,000 person-years,^8^ and UK axial SpA prevalence of 0·3%.^37^ Whilst our estimates are at the lower end of these statistics, a North American EHR study using similar methods – an algorithm to identify patients with a diagnosis of axial SpA that required serial diagnosis codes – estimated axial SpA prevalence in 2009 to be 0·107%,^39^ similar to our English 2009 prevalence (0·09%). Overall, our incidence and prevalence estimates are broadly consistent with published studies, supporting the accuracy of our approach to ascertain patients with diagnoses of RA, PsA, and axial SpA.

Our study's strengths are its systematic approach to determining diagnosis code lists, evaluation of large patient numbers across England (optimising generalisability), and assessment of the three commonest forms of inflammatory arthritis simultaneously. It has several limitations. First, as we could not access unstructured text (generally unavailable for research) or participant's secondary care records we could not be entirely certain patients had inflammatory arthritis. However, we updated previously validated algorithms demonstrated in other primary care EHR databases to perform well at correctly identifying patients with these conditions, our approaches produced incidence/prevalence results broadly consistent with published studies and identified expected age and sex distributions. Second, there was an absence of data on secondary care prescribed biologic and targeted synthetic DMARDs, although as synthetic DMARD prescriptions were only considered in the RA algorithm, and within England it is a prerequisite that patients with RA receive at least two synthetic DMARDs before progressing to receive a biologic/targeted synthetic DMARD, we do not consider this impacted on the RA algorithm's performance. Third, our diagnosis algorithms will inevitably misclassify some people. This is particularly relevant to those with axial SpA, with only 56·6 to 62·7% of people with an axial SpA Read/SNOMED code having a second code after ≥seven days. This is very similar to the proportion (57·1%) in the THIN-based diagnosis algorithm development study. Whilst requiring a second code optimises the PPV, it will inevitably reduce sensitivity and the decision on whether to use this algorithm in future studies of axial SpA depends on their context.

In conclusion, the algorithms we used to ascertain patients with diagnoses of RA, PsA, and axial SpA led to incidence and prevalence estimates broadly consistent with published studies, supporting their use in future research, and demonstrating that the recording of diagnoses of these three conditions is increasingly prevalent in England, particularly in those aged ≥65 years. This latter finding underlines the importance of organising healthcare services in England to provide timely, treat-to-target inflammatory arthritis care to optimise the health of over 1% of adults, in a manner that is acceptable to older people.

## Contributors

ICS, CDM, and KPJ were involved with funding acquisition. ICS, SLH, CDM, SM, and KPJ conceptualised the study. ICS, RW, HT, SLH, CDM, SM, KPJ contributed to methodology. RW and JB undertook data curation. RW undertook data analysis. ICS, KPJ, and SM supervised the study. ICS and RW wrote the first draft of the manuscript. All authors reviewed and edited the manuscript.

## Data sharing statement

Data may be obtained from a third party and are not publicly available. The data were obtained from the CPRD. CPRD data governance does not allow us to distribute patient data to other parties. Researchers may apply for data access at http://www.CPRD.com/.

## Declaration of interests

Relevant to the present manuscript: access to CPRD data and ICS's salary was funded by an NIHR Advanced Research Fellowship award; CDM's salary is funded by the NIHR School for Primary Care Research and NIHR Applied Research Collaboration; KPJ's salary is partly funded by the NIHR Applied Research Collaboration; SM's salary is partly funded by the NIHR Applied Research Collaboration. In the last three years: ICS has received grant funding from the British Society for Rheumatology and received support for attendance at a conference from the NIHR; Keele University have received funding for CDM's salary from the MRC, AHRC, Versus Arthritis, NIHR, and BMS.

## Appendix Supplementary materials

Image, application 1

Image, application 2
