# Supplementary material for: Rheumatoid arthritis, psoriatic arthritis, and axial spondyloarthritis epidemiology in England from 2004 to 2020: An observational study using primary care electronic health record data
Source: Lancet Reg Health Eur. 2022 Oct 10;23:100519. doi: 10.1016/j.lanepe.2022.100519 (PMC9557034; doi:10.1016/j.lanepe.2022.100519)
Supplement: Supplementary file 1 [file mmc1.docx]

**Establishing the Epidemiology of Rheumatoid Arthritis, Psoriatic Arthritis, and Axial Spondyloarthritis in England using Primary Care Electronic Health Record Data: A Descriptive Observational Study**

**Supplementary Data**

**Supplementary Table 1. Details of Process for Selecting Read/SNOMED Codes for a Diagnosis of Rheumatoid Arthritis, Psoriatic Arthritis, or Axial Spondyloarthritis**

| **SNOMED Coding Overview** |
| --- |
| SNOMED CT uses “concept” and “description” IDs (1). These are integer values with an associated medical “term” providing a textual diagnosis description. Description IDs have terms representing synonyms e.g., “Heart Attack”. They map to concept IDs with terms representing the preferred description e.g., “Myocardial Infarction”. As some similar “terms” exist for the same description ID the unique diagnosis code identifier in Aurum is the “Medcode ID”. Read codes were used to code primary care diagnoses before 2018; since 2018 SNOMED codes have been used instead, with every Read code and term mapping to a SNOMED code. |
| **Read/SNOMED Code List Generation Overview** |
| To generate Read/SNOMED code lists for diagnoses of RA, PsA, and axial SpA the Aurum browser was searched by ICS for relevant substring within medical terms. Additionally, SNOMED codes mapping to existing Read code lists for RA (2) and PsA (3) were identified. Prototype Read/SNOMED code lists were reviewed by two consultant rheumatologists (ICS/SH) and two GPs (CDM/HT) leading to 89, 6, and 5 agreed Read/SNOMED codes for diagnoses of RA, PsA, and axial SpA, respectively (available in Supplementary Files). |
| **Rheumatoid Arthritis Code List Generation** |
| The Aurum medical dictionary was searched for medical terms containing the substring “rheumatoid” or “RA” (the latter as a separate word).  Medical terms addressing rheumatoid factor tests, juvenile arthritis, screening for RA, and family history of RA were removed.  Medical terms for RA of the “sacroiliac joint” (a joint unaffected by RA) and “O/E-hands-rheumatoid spindling” (a non-specific term) were also removed.  Following merging with the existing Read code list developed by Muller at al in CPRD GOLD (2), and mapping the identified concept IDs to description IDs, a list of 122 description IDs and associated Medical terms was generated for group review.  From these 33 were removed, leading to a final list of 89 codes for a diagnosis of RA. |
| **Psoriatic Arthritis Code List Generation** |
| The Aurum medical dictionary was searched for medical terms containing the substring “psoria” or “arthritis mutilans”.  Medical terms for psoriasis (without arthritis) and family history of psoriasis were removed.  Following merging with the existing Read code list developed by Ogdie et al in THIN (3), and mapping the identified concept IDs to description IDs, a list of 9 description IDs with associated medical terms was generated for group review.  From this 3 were removed, comprising “juvenile arthritis in psoriasis” (distinct to adult PsA), “arthritis mutilans” (as it was felt this could be used by GPs to describe any severely deforming form of arthritis), and “psoriatic arthritis impact of disease 9 questionnaire" (which on its own was considered insufficient evidence of having PsA). This led to a final list of 6 codes for a diagnosis of PsA. |
| **Axial Spondyloarthritis Code List Generation** |
| The Aurum medical dictionary was searched for medical terms containing the substring "ankylosing spond”, “sacroiliitis”, “sacroiliac”, “spondyloa”, “reactive arth”, “spondylitis”.  Medical terms for non-axial spondyloarthritis, infective spinal problems, non-inflammatory spinal pathologies, juvenile ankylosing spondylitis, family history of ankylosing spondylitis, and sacroiliac X-ray were excluded.  Following mapping the identified concept IDs to description IDs, a list of 12 description IDs with associated medical terms was generated for group review.  From this 7 were removed, comprising "other rheumatoid arthritis of spine" and "osteoarthritis nos, of sacro-iliac joint" (alternative diagnoses), “sacroiliitis NEC” (imaging changes alone considered insufficient for a diagnosis of axial SpA), “reactive arthropathy of the sacro-iliac joint” (considered insufficient for a diagnosis of an axial SpA, as it could be used in other settings such as back pain in the context of an infection) and the outcome measures “Bath Ankylosing Spondylitis Functional Index”, “Bath Ankylosing Spondylitis Metrology Index”, and “BASDAI - Bath Ankylosing Spondylitis Disease Activity Index” (considered, on their own, insufficient for a diagnosis of an axial SpA) leading to a final list of 5 codes for a diagnosis of axial SpA. |

**Supplementary Table 2. SNOMED/Read Codes for a Diagnosis of Rheumatoid Arthritis**

| **SNOMED CT Concept ID** | **Term** | **SNOMED CT Description ID** | **Read code V3** | **Evidence Strength** |
| --- | --- | --- | --- | --- |
| 239791005 | [x]other seropositive rheumatoid arthritis | 359291017 | nyu1100 | 1 |
| 239791005 | [x]seropositive rheumatoid arthritis, unspecified | 359291017 | nyu1g00 | 1 |
| 239791005 | seropositive rheumatoid arthritis, unspecified | 359291017 | n04x.00 | 1 |
| 239792003 | seronegative rheumatoid arthritis | 359292012 | n040p00 | 1 |
| 308143008 | seropositive errosive rheumatoid arthritis | 3517964017 | n047.00 | 1 |
| 69896004 | rheumatoid arthritis | 116082011 | n040.00 | 1 |
| 69896004 | rheumatoid arthritis nos | 889731000006111 | n040.90 | 1 |
| 909191000006103 | [rfc] rheumatoid arthritis | 909191000006119 | NA | 1 |
| 201764007 | rheumatoid arthritis of cervical spine | 309787016 | n040000 | 2 |
| 201766009 | rheumatoid arthritis of shoulder | 309789018 | n040200 | 2 |
| 201767000 | rheumatoid arthritis of sternoclavicular joint | 309790010 | n040300 | 2 |
| 201768005 | rheumatoid arthritis of acromioclavicular joint | 309791014 | n040400 | 2 |
| 201769002 | rheumatoid arthritis of elbow | 309792019 | n040500 | 2 |
| 201770001 | rheumatoid arthritis of distal radio-ulnar joint | 309793012 | n040600 | 2 |
| 201771002 | rheumatoid arthritis of wrist | 309794018 | n040700 | 2 |
| 201771002 | rheum. arth. - wrist joint | 989291000006112 | n040799 | 2 |
| 201772009 | rheum. arth. - hand joint | 989301000006113 | n040899 | 2 |
| 201772009 | rheumatoid arthritis of mcp joint | 309795017 | n040800 | 2 |
| 201773004 | rheumatoid arthritis of pip joint of finger | 309796016 | n040900 | 2 |
| 201774005 | rheumatoid arthritis of dip joint of finger | 309797013 | n040a00 | 2 |
| 201775006 | rheumatoid arthritis of hip | 309798015 | n040b00 | 2 |
| 201775006 | rheum. arth. - hip joint | 989311000006111 | n040b99 | 2 |
| 201777003 | rheumatoid arthritis of knee | 309800010 | n040d00 | 2 |
| 201777003 | rheum. arth. - knee joint | 989321000006115 | n040d99 | 2 |
| 201778008 | rheumatoid arthritis of tibio-fibular joint | 309801014 | n040e00 | 2 |
| 201779000 | rheumatoid arthritis of ankle | 309802019 | n040f00 | 2 |
| 201779000 | rheum. arth. - ankle/foot | 989331000006117 | n040f99 | 2 |
| 201780002 | rheumatoid arthritis of subtalar joint | 309803012 | n040g00 | 2 |
| 201781003 | rheumatoid arthritis of talonavicular joint | 309804018 | n040h00 | 2 |
| 201783000 | rheumatoid arthritis of 1st mtp joint | 309807013 | n040k00 | 2 |
| 201784006 | rheumatoid arthritis of lesser mtp joint | 309808015 | n040l00 | 2 |
| 201785007 | rheumatoid arthritis of ip joint of toe | 309809011 | n040m00 | 2 |
| 201791009 | flare of rheumatoid arthritis | 309816012 | n040t00 | 2 |
| 287006005 | rheumatoid arthritis - multiple joint | 426510015 | n040s00 | 2 |
| 429192004 | rheumatoid arthritis of other tarsal joint | 2696026017 | n040j00 | 2 |
| 69896004 | rheum. arth. - other joint | 889741000006118 | n040.91 | 2 |
| 69896004 | rheum. arth. - ankle/foot | 889751000006116 | n040.92 | 2 |
| 69896004 | rheum. arth. - knee joint | 889761000006119 | n040.93 | 2 |
| 69896004 | rheum. arth. - hip joint | 889771000006114 | n040.94 | 2 |
| 69896004 | rheum. arth. - hand joint | 889781000006112 | n040.95 | 2 |
| 69896004 | rheum. arth. - wrist joint | 889791000006110 | n040.96 | 2 |
| 69896004 | rheum. arth. - elbow joint | 889801000006111 | n040.97 | 2 |
| 69896004 | rheum. arth. - shoulder joint | 889811000006114 | n040.98 | 2 |
| 69896004 | rheum. arth. - multiple joint | 889821000006118 | n040.99 | 2 |
| 9631008 | other rheumatoid arthritis of spine | 16840014 | n040100 | 2 |
| 10713006 | fibrosing alveolitis associated with rheumatoid arthritis | 311496011 | n04y012 | 3 |
| 193180002 | polyneuropathy in rheumatoid arthritis | 297544012 | f371200 | 3 |
| 193250002 | myopathy due to rheumatoid arthritis | 297641010 | f396400 | 3 |
| 195136004 | rheumatoid myocarditis | 300221014 | g5y8.00 | 3 |
| 239793008 | rheumatoid arthropathy + visceral/systemic involvement nos | 359293019 | n042z00 | 3 |
| 239793008 | other rheumatoid arthropathy + visceral/systemic involvement | 359293019 | n042.00 | 3 |
| 239793008 | [x]rheumatoid arthritis+involvement/other organs or systems | 359293019 | nyu1000 | 3 |
| 28880005 | rheumatoid carditis | 48361011 | g5ya.00 | 3 |
| 33719002 | rheumatoid nodule | 485614018 | n042200 | 3 |
| 33719002 | rheumatoid nodule | 485614018 | n040r00 | 3 |
| 398640008 | caplan's syndrome | 1786506012 | n04y011 | 3 |
| 398726004 | rheumatoid lung disease | 1778239014 | n042100 | 3 |
| 398726004 | rheumatoid lung | 1786545019 | n04y000 | 3 |
| 398726004 | rheumatoid lung | 1786545019 | h570.00 | 3 |
| 400054000 | rheumatoid vasculitis | 1779323014 | n040n00 | 3 |
| 57160007 | felty's syndrome | 95067016 | n041.00 | 3 |
| 1048311000000106 | rheumatoid arthritis impact of disease questionnaire | 2640901000000118 | NA | 4 |
| 1085601000000101 | disease activity score in rheumatoid arthritis | 2719771000000115 | 38dz.00 | 4 |
| 1085601000000101 | das - disease activity score | 2719771000000115 | 38dz.11 | 4 |
| 161567008 | h/o: rheumatoid arthritis | 251794010 | 14g1.00 | 4 |
| 1875561000006103 | rheumatoid arthritis monitoring invitation | 1875561000006119 | NA | 4 |
| 1875571000006105 | rheumatoid arthritis monitoring telephone invitation | 1875571000006114 | NA | 4 |
| 1875581000006108 | rheumatoid arthritis monitoring verbal invitation | 1875581000006112 | NA | 4 |
| 1875591000006106 | rheumatoid arthritis monitoring invitation first letter | 1875591000006110 | NA | 4 |
| 1875601000006103 | rheumatoid arthritis monitoring invitation second letter | 1875601000006119 | NA | 4 |
| 1875611000006100 | rheumatoid arthritis monitoring invitation third letter | 1875611000006116 | NA | 4 |
| 1932121000006100 | 3d study - problems with rheumatoid arthritis management | 1932121000006116 | NA | 4 |
| 231041000000107 | delivery of rehabilitation for rheumatoid arthritis | 371261000000111 | 7p20300 | 4 |
| 275902004 | rheumatoid arthrit. monitoring | 411871019 | 66h..13 | 4 |
| 441870009 | disease activity score using 28 joint count | 2817437013 | 2hj..00 | 4 |
| 441870009 | disease activity score 28 joint in rheumatoid arthritis | 2817437013 | 38dz000 | 4 |
| 69896004 | [x]other specified rheumatoid arthritis | 116082011 | nyu1200 | 4 |
| 69896004 | rheumatoid arthritis and other inflammatory polyarthropathy | 116082011 | n04..00 | 4 |
| 805941000000105 | exception reporting: rheumatoid arthritis quality indicators | 1801791000000117 | 9hr..00 | 4 |
| 805961000000106 | except rheumatoid arthritis quality indicator: pt unsuitable | 1801831000000112 | 9hr0.00 | 4 |
| 805981000000102 | except rheumatoid arthritis qual indicator: informed dissent | 1801871000000114 | 9hr1.00 | 4 |
| 84017003 | rheumatoid bursitis | 139318017 | n040q00 | 4 |
| 847261000000104 | rheumatoid arthritis annual review | 2196791000000119 | 66hb000 | 4 |
| 882321000000105 | rheumatoid arthritis monitoring invitation | 2275501000000119 | 9mm..00 | 4 |
| 882401000000106 | rheumatoid arthritis monitoring invitation first letter | 2275671000000111 | 9mm0.00 | 4 |
| 882421000000102 | rheumatoid arthritis monitoring invitation second letter | 2275711000000112 | 9mm1.00 | 4 |
| 882441000000109 | rheumatoid arthritis monitoring invitation third letter | 2275751000000111 | 9mm2.00 | 4 |
| 882461000000105 | rheumatoid arthritis monitoring verbal invitation | 2275791000000115 | 9mm3.00 | 4 |
| 882481000000101 | rheumatoid arthritis monitoring telephone invitation | 2275831000000110 | 9mm4.00 | 4 |

**Supplementary Table 3. SNOMED/Read Codes for a Diagnosis of Psoriatic Arthritis**

| **SNOMED CT Concept ID** | **Term** | **SNOMED CT Description ID** | **Read code V3** |
| --- | --- | --- | --- |
| 33339001 | psoriatic arthritis | 55628018 | m160.11 |
| 33339001 | psoriatic arthropathy | 55627011 | m160.00 |
| 33339001 | psoriatic arthropathy nos | 55627011 | m160z00 |
| 33339001 | [x]other psoriatic arthropathies | 55626019 | nyu1300 |
| 200956002 | psoriasis spondylitica | 308727011 | m160000 |
| 239812005 | distal interphalangeal psoriatic arthropathy | 359321011 | m160100 |

**Supplementary Table 4. SNOMED/Read Codes for a Diagnosis of Axial Spondyloarthritis**

| **SNOMED CT Concept ID** | **Term** | **SNOMED CT Description ID** | **Read code V3** |
| --- | --- | --- | --- |
| 9631008 | ankylosing spondylitis | 16833013 | n100.00 |
| 9631008 | marie - strumpell spondylitis | 1235977013 | n100.11 |
| 1898101000006106 | axial spondyloarthritis | 1898101000006110 | NA |
| 723116002 | axial spondyloarthritis | 3334645019 | n11f.00 |
| 713777005 | non-radiographic axial spondyloarthritis | 3297625017 | NA |

**Supplementary Table 5. Main Synthetic DMARD Indications**

| **Drug** | **Key Indications** |
| --- | --- |
| Azathioprine | RA, PsA, other SpA, JIA, connective tissue diseases, SLE, transplantation, IBD. |
| Ciclosporin | RA, PsA, other SpA, transplantation, IBD, psoriasis. |
| Hydroxychloroquine | RA, other SpA, SLE. |
| Leflunomide | RA, PsA, other SpA. |
| Methotrexate | RA, PsA, other SpA, IBD, psoriasis, JIA, vasculitis. |
| Penicillamine | RA. |
| Sodium Aurothiomalate | RA. |
| Sulfasalazine | RA, PsA, other SpA, IBD, JIA. |

**Supplementary Table 6. Substring text used to search for relevant Read/SNOMED codes for alternative DMARD indications**

| **Alternative DMARD Indication** | **Substring Search Text** |
| --- | --- |
| Other spondyloarthropathies | "spondyloa”, “reactive arth”, “reiter”, “spondylitis”. |
| JIA | “juvenile”, “stills”, “JIA”. |
| SLE | “lupus”, “libman”, “SLE”. |
| Any vasculitis | “temporal arteritis”, “giant cell arteritis”, “takayasu”, “polyarteritis”, “Kawasaki”, “acute febrile mucocutaneous lymph node”, “polyangiitis”, “bechet”, “vasculitis”, “cryoglobulin”, “coogan”, “henoch”, “churg”, “Wegener”. |
| ANCA-associated vasculitis | “Wegener’s granulomatosis”, “granulomatosis with polyangiitis”, “churg-strauss vasculitis”. |
| Giant cell arteritis | "temporal arteritis", "cranial arteritis", "giant cell arteritis", "giant cell arteritis nos","[x]other giant cell arteritis","giant cell arteritis with polymyalgia rheumatica","aortic arch arteritis","aortitis","[x]aortitis in diseases classified elsewhere". |
| Still’s disease | “still’s”. |
| Uveitis | “uveitis”. |
| Psoriasis | “psoriasis”. |
| IBD | “crohn”, “colitis”, “inflammatory bowel”. |
| Transplantation | “transplant”. |
| Connective tissue disease | “myositis”, “sjogren”, “scleroderma”, “systemic sclerosis”, “connective tissue”. |
| Non-Hodgkin’s lymphoma and chronic lymphocytic leukaemia | “non-hodgkin”, “chronic lymphocytic leukaemia”. |

Read/SNOMED codes and associated Medical terms for these diagnoses were identified from the Aurum medical dictionary by searching for medical terms containing relevant substring. Due to the large number of potential terms for “vasculitis”, codes were also included from the alternative diagnosis list reported by Muller et al (2). Irrelevant identified codes were excluded.

**Supplementary Table 7. Proportion Meeting Algorithm for RA Diagnosis**

| **Year** | **Number Patients with Read/SNOMED Code(s)** | **Number (%) Meeting Algorithm** | **Number (%) Meeting Criteria 1** | **Number (%) Meeting Criteria 2** | **Number (%) With ≥2 Read/SNOMED Codes** | **Number (%) Without Alternative Diagnosis** | **Number (%) With Read/SNOMED Code from Groups 1/2** |
| --- | --- | --- | --- | --- | --- | --- | --- |
| 2004 | 63,781 | 51,454 (80.7) | 40,013 (62.7) | 47,129 (73.9) | 48,043 (75.3) | 63,164 (99.0) | 61,038 (95.7) |
| 2005 | 65,745 | 53,754 (81.8) | 41,883 (63.7) | 49,639 (75.5) | 50,643 (77.0) | 65,110 (99.0) | 62,895 (95.7) |
| 2006 | 67,366 | 55,982 (83.1) | 43,785 (65.0) | 52,002 (77.2) | 53,059 (78.8) | 66,697 (99.0) | 64,441 (95.7) |
| 2007 | 68,645 | 58,046 (84.6) | 45,606 (66.4) | 54,231 (79.0) | 55,384 (80.7) | 67,932 (99.0) | 65,647 (95.6) |
| 2008 | 69,598 | 59,925 (86.1) | 47,341 (68.0) | 56,372 (81.0) | 57,582 (82.7) | 68,869 (99.0) | 66,561 (95.6) |
| 2009 | 70,919 | 62,158 (87.7) | 49,410 (69.7) | 58,749 (82.8) | 60,021 (84.6) | 70,157 (98.9) | 67,856 (95.7) |
| 2010 | 72,539 | 64,533 (89.0) | 51,493 (71.0) | 61,332 (84.6) | 62,698 (86.4) | 71,754 (98.9) | 69,336 (95.6) |
| 2011 | 73,810 | 66,679 (90.3) | 53,242 (72.1) | 63,770 (86.4) | 65,215 (88.4) | 73,011 (98.9) | 70,377 (95.4) |
| 2012 | 75,538 | 69,284 (91.7) | 55,264 (73.2) | 66,766 (88.4) | 68,319 (90.4) | 74,744 (99.0) | 71,755 (95.0) |
| 2013 | 80,737 | 73,081 (90.5) | 58,198 (72.1) | 70,510 (87.3) | 73,938 (91.6) | 79,775 (98.8) | 73,559 (91.1) |
| 2014 | 84,952 | 76,747 (90.3) | 61,228 (72.1) | 74,214 (87.4) | 78,997 (93.0) | 83,824 (98.7) | 75,256 (88.6) |
| 2015 | 89,186 | 80,067 (89.8) | 64,286 (72.1) | 77,065 (86.4) | 82,694 (92.7) | 87,867 (98.5) | 77,848 (87.3) |
| 2016 | 93,117 | 83,187 (89.3) | 67,184 (72.2) | 79,764 (85.7) | 86,091 (92.5) | 91,643 (98.4) | 80,507 (86.5) |
| 2017 | 97,429 | 86,533 (88.8) | 70,177 (72.0) | 82,607 (84.8) | 89,551 (91.9) | 95,786 (98.3) | 83,421 (85.6) |
| 2018 | 103,912 | 89,872 (86.5) | 73,021 (70.3) | 85,210 (82.0) | 93,602 (90.1) | 101,689 (97.9) | 86,186 (82.9) |
| 2019 | 108,183 | 92,335 (85.4) | 75,033 (69.4) | 86,912 (80.3) | 95,786 (88.5) | 105,762 (97.8) | 88,333 (81.7) |
| 2020 | 107,168 | 90,551 (84.5) | 73,663 (68.7) | 84,476 (78.8) | 93,224 (87.0) | 104,712 (97.7) | 87,217 (81.4) |

**Supplementary Table 8. Number of Patients with Read/SNOMED Codes for Psoriatic Arthritis Diagnosis**

| **Year** | **Number Patients with Read/SNOMED Code(s)** |
| --- | --- |
| 2004 | 13,594 |
| 2005 | 14,634 |
| 2006 | 15,635 |
| 2007 | 16,656 |
| 2008 | 17,675 |
| 2009 | 18,911 |
| 2010 | 20,182 |
| 2011 | 21,431 |
| 2012 | 22,642 |
| 2013 | 24,020 |
| 2014 | 25,584 |
| 2015 | 27,198 |
| 2016 | 28,744 |
| 2017 | 30,437 |
| 2018 | 32,157 |
| 2019 | 33,636 |
| 2020 | 32,770 |

**Supplementary Table 9. Proportion Meeting Algorithm for Axial SpA Diagnosis**

| **Year** | **Number Patients with Read/SNOMED Code(s)** | **Number (%) With ≥2 Read/SNOMED Codes** **≥7 Days Apart** |
| --- | --- | --- |
| 2004 | 13,210 | 7,468 (56.5) |
| 2005 | 13,632 | 7,810 (57.3) |
| 2006 | 14,043 | 8,169 (58.2) |
| 2007 | 14,427 | 8,459 (58.6) |
| 2008 | 14,807 | 8,789 (59.4) |
| 2009 | 15,182 | 9,097 (59.9) |
| 2010 | 15,563 | 9,382 (60.3) |
| 2011 | 15,870 | 9,615 (60.6) |
| 2012 | 16,349 | 9,970 (61.0) |
| 2013 | 16,781 | 10,329 (61.6) |
| 2014 | 17,253 | 10,717 (62.1) |
| 2015 | 17,950 | 11,224 (62.5) |
| 2016 | 18,730 | 11,745 (62.7) |
| 2017 | 19,647 | 12,315 (62.7) |
| 2018 | 20,595 | 12,855 (62.4) |
| 2019 | 21,484 | 13,272 (61.8) |
| 2020 | 21,343 | 12,938 (60.6) |

**Supplementary Table 10. Annual Age and Sex of Patients Meeting Rheumatoid Arthritis Diagnosis Algorithm**

| **Year** | **Age, mean (SD)** | **Female, n (%)** |
| --- | --- | --- |
| 2004 | 64.63 (13.95) | 36,624 (71.18) |
| 2005 | 64.81 (14.06) | 38,293 (71.24) |
| 2006 | 65.03 (14.14) | 39,865 (71.21) |
| 2007 | 65.22 (14.24) | 41,255 (71.07) |
| 2008 | 65.41 (14.32) | 42,605 (71.10) |
| 2009 | 65.51 (14.43) | 44,119 (70.98) |
| 2010 | 65.63 (14.51) | 45,821 (71.00) |
| 2011 | 65.73 (14.59) | 47,383 (71.06) |
| 2012 | 65.88 (14.70) | 49,166 (70.96) |
| 2013 | 65.92 (14.85) | 51,863 (70.97) |
| 2014 | 65.84 (14.88) | 54,416 (70.90) |
| 2015 | 65.76 (14.93) | 56,723 (70.84) |
| 2016 | 65.81 (14.97) | 58,921 (70.83) |
| 2017 | 65.85 (14.97) | 61,174 (70.69) |
| 2018 | 65.88 (14.98) | 63,346 (70.48) |
| 2019 | 66.01 (15.00) | 65,089 (70.49) |
| 2020 | 66.22 (14.96) | 63,865 (70.53) |

**Supplementary Table 11. Annual Age and Sex of Patients with a Read/SNOMED Code for Psoriatic Arthritis Diagnosis**

| **Year** | **Age, mean (SD)** | **Female, n (%)** |
| --- | --- | --- |
| 2004 | 53.87 (14.21) | 6,494 (47.77) |
| 2005 | 54.10 (14.27) | 6,997 (47.81) |
| 2006 | 54.40 (14.22) | 7,482 (47.85) |
| 2007 | 54.76 (14.19) | 7,986 (47.95) |
| 2008 | 54.99 (14.27) | 8,447 (47.79) |
| 2009 | 55.20 (14.29) | 9,054 (47.88) |
| 2010 | 55.37 (14.29) | 9,719 (48.16) |
| 2011 | 55.61 (14.29) | 10,344 (48.27) |
| 2012 | 55.92 (14.30) | 11,060 (48.85) |
| 2013 | 56.13 (14.37) | 11,810 (49.17) |
| 2014 | 56.36 (14.37) | 12,631 (49.37) |
| 2015 | 56.56 (14.43) | 13,520 (49.71) |
| 2016 | 56.82 (14.44) | 14,334 (49.87) |
| 2017 | 57.01 (14.49) | 15,286 (50.22) |
| 2018 | 57.18 (14.56) | 16,209 (50.66) |
| 2019 | 57.41 (14.63) | 17,192 (51.11) |
| 2020 | 57.95 (14.61) | 16,742 (51.09) |

**Supplementary Table 12. Annual Age and Sex of Patients Meeting Axial Spondyloarthritis Diagnosis Algorithm**

| **Year** | **Age, mean (SD)** | **Female, n (%)** |
| --- | --- | --- |
| 2004 | 51.67 (13.49) | 1,595 (21.36) |
| 2005 | 52.06 (13.65) | 1,701 (21.78) |
| 2006 | 52.35 (13.69) | 1,758 (21.52) |
| 2007 | 52.80 (13.79) | 1,847 (21.83) |
| 2008 | 53.16 (13.92) | 1,939 (22.06) |
| 2009 | 53.41 (14.02) | 2,021 (22.22) |
| 2010 | 53.77 (14.15) | 2,124 (22.64) |
| 2011 | 53.96 (14.27) | 2,215 (23.04) |
| 2012 | 54.19 (14.38) | 2,338 (23.45) |
| 2013 | 54.34 (14.49) | 2,446 (23.68) |
| 2014 | 54.50 (14.66) | 2,585 (24.12) |
| 2015 | 54.44 (14.88) | 2,817 (25.10) |
| 2016 | 54.53 (15.02) | 3,042 (25.90) |
| 2017 | 54.66 (15.19) | 3,280 (26.63) |
| 2018 | 54.80 (15.27) | 3,537 (27.51) |
| 2019 | 54.91 (15.30) | 3,744 (28.21) |
| 2020 | 55.28 (15.25) | 3,747 (28.96) |

**Supplementary Table 13. Annual Incidence of Rheumatoid Arthritis Diagnoses, Overall and Stratified by Sex**

| **Year** | | **Overall Crude** | **Overall Age-Sex Standardised** | **In Males** | **In Females** |
| --- | --- | --- | --- | --- | --- |
| 2004 | 39.994 (38.644, 41.39) | | 42.441 (42.294, 42.588) | 25.382 (23.882, 26.976) | 54.621 (52.398, 56.939) |
| 2005 | 38.320 (37.009, 39.678) | | 40.42 (40.278, 40.562) | 23.318 (21.892, 24.837) | 53.359 (51.177, 55.634) |
| 2006 | 34.961 (33.720, 36.249) | | 37.018 (36.883, 37.153) | 22.558 (21.167, 24.039) | 47.397 (45.359, 49.527) |
| 2007 | 34.469 (33.246, 35.738) | | 36.333 (36.201, 36.465) | 22.408 (21.033, 23.873) | 46.567 (44.563, 48.661) |
| 2008 | 35.613 (34.380, 36.89) | | 37.608 (37.474, 37.741) | 21.994 (20.643, 23.434) | 49.258 (47.213, 51.392) |
| 2009 | 39.279 (37.992, 40.610) | | 41.313 (41.175, 41.452) | 25.884 (24.425, 27.432) | 52.681 (50.58, 54.87) |
| 2010 | 37.727 (36.473, 39.025) | | 39.605 (39.471, 39.74) | 23.287 (21.912, 24.748) | 52.17 (50.091, 54.335) |
| 2011 | 38.268 (37.010, 39.568) | | 39.97 (39.836, 40.104) | 24.224 (22.826, 25.708) | 52.274 (50.203, 54.43) |
| 2012 | 39.393 (38.124, 40.704) | | 40.984 (40.85, 41.119) | 25.962 (24.521, 27.487) | 52.779 (50.712, 54.931) |
| 2013 | 52.115 (50.648, 53.624) | | 53.976 (53.821, 54.131) | 33.96 (32.302, 35.704) | 70.201 (67.802, 72.684) |
| 2014 | 50.535 (49.089, 52.024) | | 52.316 (52.163, 52.468) | 33.799 (32.145, 35.538) | 67.325 (64.97, 69.766) |
| 2015 | 51.482 (50.034, 52.972) | | 53.059 (52.907, 53.211) | 33.527 (31.894, 35.244) | 69.519 (67.144, 71.978) |
| 2016 | 49.128 (47.729, 50.567) | | 50.565 (50.419, 50.711) | 32.321 (30.737, 33.988) | 66.029 (63.74, 68.399) |
| 2017 | 49.466 (48.079, 50.893) | | 51.027 (50.882, 51.173) | 32.327 (30.762, 33.972) | 66.752 (64.476, 69.109) |
| 2018 | 51.466 (50.066, 52.906) | | 53.122 (52.976, 53.269) | 35.026 (33.412, 36.717) | 68.073 (65.796, 70.429) |
| 2019 | 49.103 (47.737, 50.509) | | 50.807 (50.664, 50.951) | 32.308 (30.761, 33.932) | 66.13 (63.886, 68.452) |
| 2020 | 29.395 (28.337, 30.492) | | 30.488 (30.376, 30.6) | 19.637 (18.435, 20.918) | 39.277 (37.55, 41.084) |

Data are incidence rates per 100,000 person-years with 95% confidence intervals in brackets.

**Supplementary Table 14. Annual Incidence of Rheumatoid Arthritis Diagnoses Stratified by Age Categories**

| **Year** | **<25** | **≥25 to <35** | **≥35 to <45** | **≥45 to <55** | **≥55 to <65** | **≥65 to <75** | **≥75** |
| --- | --- | --- | --- | --- | --- | --- | --- |
| **2004** | 2.777 (1.828, 4.217) | 9.805 (8.298, 11.585) | 21.113 (19.041, 23.41) | 38.267 (35.104, 41.715) | 75.138 (70.368, 80.232) | 88.794 (82.704, 95.332) | 66.866 (61.596, 72.587) |
| **2005** | 3.575 (2.485, 5.145) | 10.308 (8.76, 12.13) | 20.956 (18.905, 23.23) | 44.982 (41.582, 48.659) | 66.627 (62.196, 71.374) | 76.377 (70.746, 82.455) | 62.938 (57.865, 68.455) |
| **2006** | 3.337 (2.304, 4.833) | 8.864 (7.439, 10.563) | 19.487 (17.525, 21.669) | 34.634 (31.708, 37.83) | 60.076 (55.919, 64.543) | 76.681 (71.044, 82.765) | 62.059 (57.062, 67.495) |
| **2007** | 5.667 (4.283, 7.498) | 9.933 (8.421, 11.715) | 19.836 (17.86, 22.03) | 37.531 (34.525, 40.8) | 59.493 (55.381, 63.91) | 70.773 (65.407, 76.578) | 54.361 (49.737, 59.415) |
| **2008** | 3.932 (2.823, 5.477) | 11.024 (9.437, 12.878) | 20.572 (18.558, 22.804) | 37.356 (34.405, 40.561) | 62.826 (58.622, 67.331) | 76.232 (70.724, 82.169) | 53.743 (49.171, 58.74) |
| **2009** | 6.408 (4.954, 8.289) | 11.381 (9.779, 13.245) | 22.681 (20.553, 25.028) | 40.605 (37.574, 43.882) | 65.019 (60.75, 69.588) | 82.647 (76.986, 88.725) | 66.218 (61.142, 71.715) |
| **2010** | 4.354 (3.194, 5.936) | 11.9 (10.274, 13.783) | 20.555 (18.522, 22.812) | 42.726 (39.658, 46.031) | 64.574 (60.339, 69.106) | 76.128 (70.736, 81.93) | 59.577 (54.796, 64.775) |
| **2011** | 5.152 (3.882, 6.836) | 11.75 (10.145, 13.609) | 23.884 (21.665, 26.331) | 39.791 (36.866, 42.948) | 62.772 (58.592, 67.25) | 78.803 (73.408, 84.593) | 61.774 (56.934, 67.026) |
| **2012** | 5.09 (3.836, 6.755) | 11.837 (10.245, 13.678) | 25.255 (22.954, 27.786) | 40.579 (37.653, 43.732) | 64.203 (59.96, 68.745) | 81.171 (75.816, 86.903) | 63.179 (58.313, 68.452) |
| **2013** | 6.433 (4.995, 8.285) | 18.373 (16.365, 20.626) | 29.617 (27.08, 32.392) | 57.342 (53.842, 61.071) | 85.956 (81.017, 91.196) | 98.247 (92.393, 104.471) | 86.516 (80.731, 92.716) |
| **2014** | 7.757 (6.157, 9.772) | 14.526 (12.754, 16.544) | 31.1 (28.477, 33.964) | 53.309 (49.936, 56.91) | 83.809 (78.941, 88.976) | 99.413 (93.565, 105.627) | 81.416 (75.774, 87.477) |
| **2015** | 8.331 (6.673, 10.401) | 16.405 (14.527, 18.525) | 31.025 (28.416, 33.873) | 56.167 (52.727, 59.831) | 83.953 (79.152, 89.045) | 97.628 (91.916, 103.694) | 81.755 (76.13, 87.795) |
| **2016** | 6.585 (5.144, 8.429) | 14.797 (13.042, 16.788) | 30.973 (28.383, 33.798) | 52.712 (49.412, 56.233) | 76.485 (71.99, 81.261) | 94.761 (89.217, 100.65) | 82.731 (77.113, 88.757) |
| **2017** | 6.822 (5.369, 8.668) | 14.392 (12.689, 16.325) | 31.389 (28.809, 34.201) | 51.806 (48.552, 55.278) | 77.314 (72.871, 82.027) | 93.986 (88.515, 99.795) | 87.669 (81.943, 93.795) |
| **2018** | 8.037 (6.464, 9.993) | 15.435 (13.69, 17.402) | 31.142 (28.601, 33.91) | 55.766 (52.39, 59.36) | 80.794 (76.322, 85.528) | 97.477 (91.939, 103.348) | 89.216 (83.499, 95.324) |
| **2019** | 6.727 (5.304, 8.532) | 13.781 (12.14, 15.644) | 26.679 (24.342, 29.24) | 52.267 (48.971, 55.786) | 82.477 (77.998, 87.214) | 90.559 (85.2, 96.257) | 89.141 (83.47, 95.198) |
| **2020** | 4.167 (3.08, 5.639) | 8.578 (7.306, 10.073) | 18.164 (16.259, 20.292) | 31.553 (28.976, 34.36) | 47.442 (44.08, 51.06) | 57.156 (52.889, 61.768) | 49.134 (44.929, 53.732) |

Data are incidence rates per 100,000 person-years with 95% confidence intervals in brackets.

**Supplementary Table 15. Annual Incidence of Psoriatic Arthritis Diagnoses, Overall and Stratified by Sex**

| **Year** | | **Overall Crude** | **Overall Age-Sex Standardised** | **In Males** | **In Females** |
| --- | --- | --- | --- | --- | --- |
| 2004 | 12.214 (11.480, 12.994) | | 12.164 (12.088, 12.24) | 12.696 (11.650, 13.837) | 11.734 (10.731, 12.829) |
| 2005 | 11.946 (11.225, 12.712) | | 11.774 (11.7, 11.848) | 11.980 (10.972, 13.081) | 11.912 (10.908, 13.008) |
| 2006 | 10.829 (10.149, 11.553) | | 10.697 (10.628, 10.767) | 11.216 (10.249, 12.274) | 10.443 (9.513, 11.463) |
| 2007 | 11.416 (10.723, 12.153) | | 11.28 (11.209, 11.351) | 11.621 (10.644, 12.688) | 11.211 (10.254, 12.258) |
| 2008 | 12.357 (11.642, 13.117) | | 12.177 (12.104, 12.25) | 13.061 (12.031, 14.180) | 11.657 (10.688, 12.714) |
| 2009 | 13.058 (12.326, 13.832) | | 12.861 (12.786, 12.935) | 13.616 (12.570, 14.750) | 12.503 (11.504, 13.587) |
| 2010 | 13.336 (12.601, 14.115) | | 13.235 (13.159, 13.31) | 12.930 (11.916, 14.029) | 13.741 (12.699, 14.869) |
| 2011 | 13.815 (13.070, 14.603) | | 13.664 (13.588, 13.74) | 13.789 (12.745, 14.918) | 13.842 (12.800, 14.968) |
| 2012 | 13.548 (12.814, 14.324) | | 13.505 (13.429, 13.58) | 12.460 (11.476, 13.529) | 14.626 (13.561, 15.774) |
| 2013 | 15.251 (14.469, 16.076) | | 15.138 (15.058, 15.218) | 14.424 (13.358, 15.574) | 16.071 (14.950, 17.277) |
| 2014 | 15.561 (14.770, 16.395) | | 15.388 (15.307, 15.468) | 14.806 (13.726, 15.970) | 16.314 (15.181, 17.532) |
| 2015 | 16.691 (15.877, 17.546) | | 16.574 (16.491, 16.657) | 14.986 (13.908, 16.147) | 18.392 (17.195, 19.671) |
| 2016 | 15.885 (15.101, 16.710) | | 15.716 (15.636, 15.796) | 15.018 (13.952, 16.166) | 16.751 (15.624, 17.960) |
| 2017 | 16.764 (15.967, 17.600) | | 16.613 (16.532, 16.694) | 15.596 (14.522, 16.750) | 17.934 (16.778, 19.169) |
| 2018 | 16.974 (16.180, 17.806) | | 16.861 (16.781, 16.942) | 14.605 (13.577, 15.710) | 19.332 (18.143, 20.600) |
| 2019 | 17.238 (16.439, 18.076) | | 17.136 (17.055, 17.218) | 15.753 (14.686, 16.897) | 18.733 (17.562, 19.981) |
| 2020 | 5.581 (5.132, 6.069) | | 5.516 (5.47, 5.563) | 5.312 (4.705, 5.997) | 5.852 (5.211, 6.571) |

Data are incidence rates per 100,000 person-years with 95% confidence intervals in brackets.

**Supplementary Table 16. Annual Incidence of Psoriatic Arthritis Diagnoses Stratified by Age Categories**

| **Year** | **<25** | **≥25 to <35** | **≥35 to <45** | **≥45 to <55** | **≥55 to <65** | **≥65 to <75** | **≥75** |
| --- | --- | --- | --- | --- | --- | --- | --- |
| **2004** | 3.155 (2.132, 4.669) | 8.664 (7.256, 10.347) | 13.475 (11.842, 15.334) | 16.408 (14.386, 18.715) | 19.607 (17.254, 22.281) | 13.686 (11.436, 16.38) | 5.533 (4.17, 7.342) |
| **2005** | 3.082 (2.082, 4.561) | 9.167 (7.714, 10.894) | 14.979 (13.262, 16.919) | 15.209 (13.289, 17.406) | 17.516 (15.324, 20.021) | 11.376 (9.342, 13.853) | 6.25 (4.799, 8.141) |
| **2006** | 3.098 (2.109, 4.55) | 7.089 (5.827, 8.624) | 11.933 (10.42, 13.665) | 16.666 (14.677, 18.923) | 17.707 (15.525, 20.197) | 9.057 (7.265, 11.291) | 4.584 (3.375, 6.226) |
| **2007** | 3.122 (2.141, 4.553) | 7.464 (6.17, 9.03) | 13.004 (11.424, 14.802) | 17.791 (15.762, 20.081) | 17.254 (15.114, 19.698) | 11.283 (9.275, 13.726) | 4.063 (2.944, 5.608) |
| **2008** | 3.033 (2.08, 4.423) | 9.564 (8.095, 11.301) | 14.82 (13.127, 16.731) | 17.934 (15.928, 20.192) | 17.664 (15.51, 20.118) | 11.658 (9.638, 14.103) | 5.104 (3.835, 6.793) |
| **2009** | 5.302 (3.996, 7.036) | 10.491 (8.959, 12.286) | 16.252 (14.468, 18.257) | 18.088 (16.106, 20.315) | 18.123 (15.944, 20.6) | 11.744 (9.742, 14.157) | 4.414 (3.25, 5.994) |
| **2010** | 3.156 (2.193, 4.542) | 9.758 (8.297, 11.476) | 15.607 (13.849, 17.588) | 18.411 (16.438, 20.621) | 20.721 (18.391, 23.346) | 14.229 (12.02, 16.843) | 4.902 (3.672, 6.544) |
| **2011** | 3.756 (2.697, 5.231) | 10.492 (8.982, 12.256) | 16.127 (14.323, 18.158) | 20.656 (18.582, 22.962) | 20.408 (18.093, 23.019) | 13.12 (11.04, 15.591) | 4.626 (3.443, 6.216) |
| **2012** | 4.135 (3.021, 5.66) | 10.676 (9.169, 12.43) | 16.364 (14.533, 18.425) | 17.523 (15.64, 19.634) | 19.151 (16.905, 21.694) | 15.043 (12.852, 17.608) | 6.433 (5.015, 8.251) |
| **2013** | 3.752 (2.694, 5.225) | 11.583 (10.012, 13.399) | 19.275 (17.25, 21.537) | 20.854 (18.788, 23.147) | 23.176 (20.689, 25.963) | 13.611 (11.553, 16.035) | 6.778 (5.306, 8.66) |
| **2014** | 3.878 (2.797, 5.376) | 10.873 (9.356, 12.637) | 18.829 (16.815, 21.085) | 23.764 (21.551, 26.205) | 21.615 (19.222, 24.307) | 15.282 (13.107, 17.817) | 6.536 (5.086, 8.401) |
| **2015** | 5.446 (4.139, 7.166) | 12.36 (10.745, 14.217) | 19.851 (17.788, 22.153) | 23.107 (20.942, 25.495) | 25.098 (22.545, 27.939) | 15.288 (13.143, 17.784) | 7.843 (6.245, 9.849) |
| **2016** | 3.762 (2.713, 5.215) | 11.905 (10.342, 13.703) | 16.94 (15.055, 19.061) | 24.757 (22.532, 27.202) | 21.589 (19.272, 24.185) | 16.69 (14.473, 19.248) | 7.614 (6.053, 9.577) |
| **2017** | 4.275 (3.16, 5.785) | 13.136 (11.513, 14.987) | 20.178 (18.132, 22.455) | 23.639 (21.478, 26.018) | 22.576 (20.244, 25.177) | 18.287 (15.978, 20.928) | 7.035 (5.557, 8.907) |
| **2018** | 5.257 (4.016, 6.881) | 13.461 (11.839, 15.305) | 19.188 (17.217, 21.384) | 25.444 (23.2, 27.904) | 23.731 (21.374, 26.348) | 16.752 (14.564, 19.269) | 6.379 (4.993, 8.15) |
| **2019** | 4.154 (3.07, 5.621) | 12.965 (11.377, 14.774) | 20.692 (18.647, 22.96) | 24.567 (22.344, 27.011) | 25.084 (22.679, 27.745) | 17.443 (15.196, 20.022) | 7.355 (5.865, 9.223) |
| **2020** | 2.083 (1.358, 3.194) | 4.43 (3.543, 5.538) | 7.648 (6.449, 9.071) | 8.14 (6.885, 9.624) | 6.813 (5.616, 8.264) | 4.583 (3.492, 6.015) | 2.502 (1.691, 3.703) |

Data are incidence rates per 100,000 person-years with 95% confidence intervals in brackets.

**Supplementary Table 17. Annual Incidence of Axial Spondyloarthritis Diagnoses, Overall and Stratified by Sex**

| **Year** | **Overall Crude** | **Overall Age-Sex Standardised** | **In Males** | **In Females** |
| --- | --- | --- | --- | --- |
| 2004 | 3.392 (3.016, 3.815) | 3.27 (3.231, 3.308) | 5.043 (4.399, 5.781) | 1.751 (1.39, 2.207) |
| 2005 | 2.719 (2.386, 3.097) | 2.621 (2.586, 2.655) | 3.883 (3.328, 4.532) | 1.56 (1.223, 1.989) |
| 2006 | 2.816 (2.480, 3.198) | 2.741 (2.706, 2.776) | 4.366 (3.778, 5.044) | 1.275 (0.976, 1.664) |
| 2007 | 2.620 (2.299, 2.986) | 2.525 (2.492, 2.559) | 3.759 (3.221, 4.387) | 1.487 (1.164, 1.9) |
| 2008 | 3.137 (2.787, 3.531) | 3.009 (2.973, 3.045) | 4.616 (4.02, 5.3) | 1.667 (1.325, 2.097) |
| 2009 | 2.800 (2.473, 3.171) | 2.722 (2.688, 2.756) | 4.102 (3.546, 4.746) | 1.508 (1.187, 1.916) |
| 2010 | 2.546 (2.236, 2.898) | 2.482 (2.449, 2.514) | 3.564 (3.051, 4.163) | 1.535 (1.212, 1.943) |
| 2011 | 2.910 (2.579, 3.284) | 2.83 (2.795, 2.864) | 4.16 (3.604, 4.801) | 1.673 (1.336, 2.095) |
| 2012 | 3.147 (2.804, 3.533) | 3.046 (3.011, 3.082) | 4.418 (3.848, 5.073) | 1.891 (1.533, 2.333) |
| 2013 | 3.596 (3.226, 4.007) | 3.498 (3.46, 3.536) | 5.155 (4.534, 5.861) | 2.055 (1.679, 2.516) |
| 2014 | 3.725 (3.348, 4.144) | 3.636 (3.597, 3.675) | 4.862 (4.26, 5.548) | 2.594 (2.166, 3.107) |
| 2015 | 4.258 (3.858, 4.701) | 4.17 (4.129, 4.212) | 4.8 (4.207, 5.476) | 3.72 (3.203, 4.319) |
| 2016 | 4.362 (3.960, 4.804) | 4.251 (4.21, 4.292) | 5.451 (4.824, 6.16) | 3.277 (2.799, 3.835) |
| 2017 | 4.528 (4.123, 4.972) | 4.408 (4.367, 4.449) | 5.48 (4.859, 6.182) | 3.576 (3.081, 4.151) |
| 2018 | 4.231 (3.845, 4.657) | 4.135 (4.096, 4.175) | 4.611 (4.05, 5.25) | 3.851 (3.341, 4.439) |
| 2019 | 4.214 (3.828, 4.638) | 4.11 (4.071, 4.15) | 4.88 (4.302, 5.535) | 3.545 (3.057, 4.111) |
| 2020 | 2.640 (2.337, 2.982) | 2.57 (2.539, 2.602) | 2.605 (2.19, 3.097) | 2.675 (2.254, 3.175) |

Data are incidence rates per 100,000 person-years with 95% confidence intervals in brackets.

**Supplementary Table 18. Annual Incidence of Axial Spondyloarthritis Diagnoses Stratified by Age Categories**

| **Year** | **<25** | **≥25 to <35** | **≥35 to <45** | **≥45 to <55** | **≥55 to <65** | **≥65 to <75** | **≥75** |
| --- | --- | --- | --- | --- | --- | --- | --- |
| **2004** | 2.272 (1.431, 3.606) | 4.617 (3.62, 5.887) | 4.629 (3.713, 5.771) | 2.808 (2.044, 3.86) | 3.921 (2.946, 5.218) | 2.185 (1.394, 3.425) | 1.383 (0.786, 2.436) |
| **2005** | 2.589 (1.688, 3.97) | 4.193 (3.249, 5.412) | 3.47 (2.694, 4.469) | 3.027 (2.237, 4.096) | 2.199 (1.508, 3.207) | 0.919 (0.46, 1.838) | 1.023 (0.532, 1.965) |
| **2006** | 1.907 (1.168, 3.112) | 4.608 (3.614, 5.876) | 3.311 (2.56, 4.283) | 2.66 (1.936, 3.656) | 2.95 (2.137, 4.072) | 2.178 (1.389, 3.414) | 0.559 (0.233, 1.343) |
| **2007** | 1.388 (0.788, 2.443) | 3.803 (2.913, 4.965) | 3.577 (2.794, 4.579) | 2.783 (2.049, 3.78) | 2.756 (1.979, 3.839) | 1.354 (0.769, 2.383) | 0.878 (0.439, 1.756) |
| **2008** | 2.134 (1.361, 3.346) | 3.882 (2.987, 5.044) | 4.655 (3.749, 5.78) | 3.349 (2.545, 4.406) | 3.033 (2.216, 4.151) | 1.869 (1.162, 3.006) | 1.086 (0.584, 2.017) |
| **2009** | 1.767 (1.083, 2.885) | 5.178 (4.135, 6.483) | 3.204 (2.466, 4.163) | 3.362 (2.568, 4.401) | 2.244 (1.56, 3.23) | 1.494 (0.885, 2.522) | 0.43 (0.162, 1.147) |
| **2010** | 1.741 (1.067, 2.843) | 3.943 (3.055, 5.09) | 3.306 (2.55, 4.286) | 2.461 (1.806, 3.356) | 2.76 (1.991, 3.827) | 1.053 (0.567, 1.957) | 1.065 (0.573, 1.98) |
| **2011** | 1.61 (0.97, 2.67) | 5.081 (4.064, 6.353) | 3.779 (2.958, 4.828) | 3.49 (2.698, 4.515) | 2.077 (1.424, 3.029) | 1.727 (1.074, 2.779) | 0.525 (0.219, 1.262) |
| **2012** | 2.121 (1.368, 3.287) | 5.274 (4.247, 6.548) | 4.553 (3.636, 5.701) | 3.596 (2.798, 4.622) | 2.246 (1.56, 3.231) | 1.745 (1.1, 2.77) | 0.207 (0.052, 0.829) |
| **2013** | 3.108 (2.16, 4.473) | 5.759 (4.684, 7.081) | 4.693 (3.748, 5.876) | 4.19 (3.321, 5.287) | 3.65 (2.742, 4.858) | 1.046 (0.579, 1.888) | 0.318 (0.102, 0.985) |
| **2014** | 2.908 (1.994, 4.241) | 5.628 (4.567, 6.936) | 4.83 (3.863, 6.039) | 4.31 (3.427, 5.422) | 3.481 (2.599, 4.662) | 2.153 (1.431, 3.24) | 0.535 (0.223, 1.286) |
| **2015** | 2.883 (1.977, 4.204) | 6.495 (5.355, 7.879) | 6.593 (5.45, 7.975) | 4.418 (3.528, 5.531) | 3.226 (2.392, 4.349) | 2.453 (1.682, 3.577) | 1.165 (0.645, 2.103) |
| **2016** | 2.821 (1.935, 4.114) | 6.934 (5.766, 8.338) | 7.852 (6.603, 9.337) | 3.939 (3.111, 4.988) | 2.82 (2.06, 3.86) | 2.468 (1.704, 3.575) | 0.834 (0.417, 1.667) |
| **2017** | 1.934 (1.234, 3.032) | 7.37 (6.181, 8.788) | 7.023 (5.859, 8.418) | 5.026 (4.083, 6.186) | 3.697 (2.824, 4.839) | 2.768 (1.958, 3.915) | 0.407 (0.153, 1.085) |
| **2018** | 2.976 (2.081, 4.256) | 5.142 (4.177, 6.329) | 6.745 (5.618, 8.098) | 5.407 (4.427, 6.605) | 3.576 (2.732, 4.68) | 2.218 (1.51, 3.257) | 0.896 (0.466, 1.722) |
| **2019** | 2.967 (2.074, 4.243) | 5.532 (4.529, 6.756) | 5.884 (4.842, 7.152) | 6.548 (5.45, 7.867) | 3.377 (2.566, 4.443) | 1.723 (1.112, 2.671) | 0.49 (0.204, 1.176) |
| **2020** | 1.686 (1.048, 2.712) | 4.085 (3.237, 5.154) | 4.402 (3.516, 5.512) | 2.966 (2.248, 3.914) | 2.046 (1.439, 2.909) | 0.704 (0.352, 1.407) | 0.6 (0.269, 1.335) |

Data are incidence rates per 100,000 person-years with 95% confidence intervals in brackets.

**Supplementary Table 19. Point Prevalence of Rheumatoid Arthritis Diagnoses, Overall and Stratified by Sex**

| **Calendar Year** | **Overall Crude** | **Overall Age-Sex Standardised** | **In Males** | **In Females** |
| --- | --- | --- | --- | --- |
| 2004 | 0.541 (0.536, 0.546) | 0.608 (0.602, 0.613) | 0.314 (0.308, 0.319) | 0.765 (0.757, 0.774) |
| 2005 | 0.559 (0.554, 0.564) | 0.628 (0.623, 0.634) | 0.323 (0.318, 0.329) | 0.791 (0.783, 0.799) |
| 2006 | 0.571 (0.566, 0.576) | 0.643 (0.638, 0.649) | 0.331 (0.325, 0.336) | 0.807 (0.799, 0.816) |
| 2007 | 0.582 (0.577, 0.587) | 0.656 (0.65, 0.661) | 0.339 (0.333, 0.344) | 0.821 (0.813, 0.829) |
| 2008 | 0.594 (0.589, 0.599) | 0.669 (0.663, 0.675) | 0.346 (0.341, 0.352) | 0.838 (0.83, 0.846) |
| 2009 | 0.608 (0.603, 0.613) | 0.684 (0.678, 0.689) | 0.355 (0.350, 0.36) | 0.857 (0.849, 0.865) |
| 2010 | 0.622 (0.618, 0.627) | 0.698 (0.693, 0.704) | 0.362 (0.357, 0.368) | 0.878 (0.87, 0.887) |
| 2011 | 0.637 (0.632, 0.642) | 0.711 (0.705, 0.716) | 0.372 (0.367, 0.378) | 0.896 (0.888, 0.905) |
| 2012 | 0.652 (0.647, 0.657) | 0.723 (0.717, 0.729) | 0.381 (0.376, 0.387) | 0.918 (0.909, 0.926) |
| 2013 | 0.693 (0.688, 0.698) | 0.767 (0.761, 0.773) | 0.405 (0.400, 0.411) | 0.976 (0.967, 0.984) |
| 2014 | 0.713 (0.708, 0.718) | 0.784 (0.778, 0.79) | 0.418 (0.412, 0.424) | 1.004 (0.995, 1.012) |
| 2015 | 0.731 (0.726, 0.737) | 0.801 (0.796, 0.807) | 0.428 (0.423, 0.434) | 1.030 (1.021, 1.038) |
| 2016 | 0.744 (0.739, 0.749) | 0.815 (0.809, 0.821) | 0.437 (0.431, 0.443) | 1.047 (1.038, 1.056) |
| 2017 | 0.755 (0.750, 0.761) | 0.828 (0.822, 0.834) | 0.445 (0.439, 0.451) | 1.062 (1.054, 1.071) |
| 2018 | 0.769 (0.764, 0.775) | 0.842 (0.837, 0.848) | 0.455 (0.449, 0.461) | 1.081 (1.072, 1.09) |
| 2019 | 0.779 (0.773, 0.784) | 0.856 (0.85, 0.862) | 0.460 (0.454, 0.466) | 1.095 (1.086, 1.104) |
| 2020 | 0.771 (0.766, 0.777) | 0.845 (0.84, 0.851) | 0.455 (0.449, 0.461) | 1.085 (1.076, 1.094) |

Data are percentages (95% confidence intervals).

**Supplementary Table 20. Point Prevalence of Rheumatoid Arthritis Diagnoses Stratified by Age Categories**

| **Year** | **<25** | **25-35** | **35-45** | **45-55** | **55-65** | **65-75** | **75+** |
| --- | --- | --- | --- | --- | --- | --- | --- |
| 2004 | 0.015 (0.013, 0.018) | 0.072 (0.068, 0.076) | 0.199 (0.193, 0.206) | 0.496 (0.485, 0.508) | 0.991 (0.974, 1.009) | 1.442 (1.417, 1.467) | 1.283 (1.259, 1.307) |
| 2005 | 0.016 (0.013, 0.018) | 0.075 (0.071, 0.079) | 0.205 (0.199, 0.212) | 0.506 (0.494, 0.517) | 1.006 (0.989, 1.023) | 1.494 (1.469, 1.519) | 1.36 (1.336, 1.384) |
| 2006 | 0.016 (0.014, 0.018) | 0.073 (0.069, 0.077) | 0.206 (0.199, 0.212) | 0.502 (0.491, 0.514) | 1.018 (1.001, 1.036) | 1.535 (1.509, 1.56) | 1.439 (1.415, 1.464) |
| 2007 | 0.018 (0.016, 0.021) | 0.075 (0.071, 0.079) | 0.209 (0.202, 0.215) | 0.502 (0.491, 0.514) | 1.028 (1.011, 1.045) | 1.566 (1.541, 1.592) | 1.485 (1.461, 1.51) |
| 2008 | 0.019 (0.016, 0.021) | 0.076 (0.072, 0.081) | 0.219 (0.213, 0.226) | 0.503 (0.492, 0.514) | 1.035 (1.018, 1.053) | 1.599 (1.574, 1.625) | 1.541 (1.515, 1.566) |
| 2009 | 0.022 (0.02, 0.025) | 0.079 (0.075, 0.084) | 0.225 (0.218, 0.232) | 0.502 (0.491, 0.513) | 1.053 (1.036, 1.071) | 1.62 (1.595, 1.646) | 1.606 (1.58, 1.632) |
| 2010 | 0.021 (0.019, 0.024) | 0.084 (0.079, 0.088) | 0.232 (0.225, 0.239) | 0.51 (0.499, 0.521) | 1.066 (1.048, 1.083) | 1.649 (1.624, 1.674) | 1.661 (1.635, 1.687) |
| 2011 | 0.022 (0.02, 0.025) | 0.086 (0.082, 0.09) | 0.238 (0.231, 0.245) | 0.512 (0.501, 0.523) | 1.078 (1.06, 1.095) | 1.658 (1.634, 1.684) | 1.73 (1.704, 1.756) |
| 2012 | 0.022 (0.02, 0.025) | 0.09 (0.086, 0.095) | 0.247 (0.24, 0.254) | 0.519 (0.508, 0.53) | 1.081 (1.064, 1.099) | 1.659 (1.635, 1.683) | 1.803 (1.777, 1.83) |
| 2013 | 0.026 (0.023, 0.029) | 0.103 (0.098, 0.108) | 0.264 (0.257, 0.272) | 0.558 (0.547, 0.569) | 1.12 (1.102, 1.138) | 1.722 (1.698, 1.747) | 1.966 (1.937, 1.994) |
| 2014 | 0.028 (0.025, 0.031) | 0.11 (0.105, 0.115) | 0.276 (0.268, 0.284) | 0.576 (0.565, 0.587) | 1.139 (1.122, 1.157) | 1.736 (1.712, 1.76) | 2.012 (1.984, 2.041) |
| 2015 | 0.028 (0.025, 0.031) | 0.116 (0.111, 0.121) | 0.288 (0.28, 0.296) | 0.593 (0.582, 0.604) | 1.15 (1.133, 1.168) | 1.762 (1.738, 1.787) | 2.066 (2.038, 2.095) |
| 2016 | 0.028 (0.025, 0.031) | 0.119 (0.114, 0.124) | 0.292 (0.284, 0.3) | 0.61 (0.599, 0.621) | 1.158 (1.141, 1.176) | 1.786 (1.762, 1.81) | 2.109 (2.08, 2.138) |
| 2017 | 0.029 (0.026, 0.032) | 0.118 (0.113, 0.123) | 0.297 (0.29, 0.305) | 0.623 (0.612, 0.635) | 1.166 (1.149, 1.183) | 1.811 (1.788, 1.835) | 2.152 (2.123, 2.18) |
| 2018 | 0.031 (0.028, 0.035) | 0.12 (0.115, 0.125) | 0.302 (0.294, 0.31) | 0.641 (0.63, 0.653) | 1.174 (1.157, 1.191) | 1.831 (1.807, 1.855) | 2.203 (2.174, 2.232) |
| 2019 | 0.031 (0.028, 0.034) | 0.122 (0.117, 0.127) | 0.305 (0.297, 0.313) | 0.654 (0.642, 0.666) | 1.189 (1.172, 1.206) | 1.859 (1.835, 1.883) | 2.243 (2.214, 2.272) |
| 2020 | 0.029 (0.026, 0.033) | 0.119 (0.114, 0.124) | 0.297 (0.289, 0.305) | 0.641 (0.629, 0.652) | 1.167 (1.15, 1.184) | 1.845 (1.82, 1.87) | 2.231 (2.202, 2.26) |

Data are percentages (95% confidence intervals).

**Supplementary Table 21. Point Prevalence of Psoriatic Arthritis Diagnoses, Overall and Stratified by Sex**

| **Year** | **Overall Crude** | **Overall Age-Sex Standardised** | **Male** | **Female** |
| --- | --- | --- | --- | --- |
| 2004 | 0.145 (0.142 ,0.147) | 0.151 (0.149, 0.154) | 0.152 (0.148, 0.156) | 0.137 (0.134, 0.141) |
| 2005 | 0.154 (0.151 ,0.156) | 0.161 (0.158, 0.164) | 0.162 (0.158, 0.165) | 0.146 (0.143, 0.15) |
| 2006 | 0.161 (0.158 ,0.164) | 0.169 (0.166, 0.172) | 0.169 (0.165, 0.173) | 0.153 (0.15, 0.157) |
| 2007 | 0.169 (0.166 ,0.171) | 0.177 (0.174, 0.18) | 0.177 (0.173, 0.181) | 0.161 (0.157, 0.164) |
| 2008 | 0.178 (0.175 ,0.181) | 0.187 (0.184, 0.19) | 0.187 (0.183, 0.191) | 0.169 (0.165, 0.173) |
| 2009 | 0.187 (0.184 ,0.19) | 0.197 (0.194, 0.2) | 0.197 (0.193, 0.201) | 0.178 (0.174, 0.182) |
| 2010 | 0.197 (0.194 ,0.2) | 0.207 (0.205, 0.21) | 0.206 (0.202, 0.21) | 0.189 (0.185, 0.192) |
| 2011 | 0.207 (0.204 ,0.21) | 0.218 (0.215, 0.221) | 0.215 (0.211, 0.22) | 0.198 (0.194, 0.202) |
| 2012 | 0.216 (0.213 ,0.218) | 0.227 (0.224, 0.23) | 0.223 (0.218, 0.227) | 0.209 (0.205, 0.213) |
| 2013 | 0.232 (0.229 ,0.235) | 0.244 (0.241, 0.247) | 0.238 (0.234, 0.242) | 0.225 (0.221, 0.229) |
| 2014 | 0.242 (0.239 ,0.245) | 0.254 (0.251, 0.257) | 0.247 (0.243, 0.252) | 0.237 (0.233, 0.241) |
| 2015 | 0.253 (0.25 ,0.256) | 0.265 (0.262, 0.268) | 0.256 (0.252, 0.261) | 0.25 (0.246, 0.255) |
| 2016 | 0.262 (0.259 ,0.265) | 0.274 (0.271, 0.278) | 0.265 (0.26, 0.269) | 0.26 (0.256, 0.264) |
| 2017 | 0.271 (0.268 ,0.274) | 0.284 (0.281, 0.287) | 0.271 (0.266, 0.275) | 0.271 (0.267, 0.276) |
| 2018 | 0.281 (0.278 ,0.284) | 0.295 (0.291, 0.298) | 0.277 (0.273, 0.282) | 0.284 (0.28, 0.289) |
| 2019 | 0.287 (0.284 ,0.291) | 0.303 (0.3, 0.306) | 0.283 (0.278, 0.287) | 0.292 (0.288, 0.297) |
| 2020 | 0.285 (0.282 ,0.288) | 0.3 (0.296, 0.303) | 0.28 (0.275, 0.284) | 0.29 (0.286, 0.295) |

Data are percentages (95% confidence intervals).

**Supplementary Table 22. Point Prevalence of Psoriatic Arthritis Diagnoses Stratified by Age Categories**

| **Year** | **<25** | **25-35** | **35-45** | **45-55** | **55-65** | **65-75** | **75+** |
| --- | --- | --- | --- | --- | --- | --- | --- |
| 2004 | 0.01 (0.008, 0.012) | 0.058 (0.055, 0.062) | 0.136 (0.131, 0.141) | 0.219 (0.212, 0.227) | 0.261 (0.252, 0.27) | 0.215 (0.205, 0.225) | 0.123 (0.116, 0.131) |
| 2005 | 0.009 (0.007, 0.011) | 0.06 (0.056, 0.064) | 0.147 (0.141, 0.152) | 0.228 (0.22, 0.235) | 0.279 (0.27, 0.288) | 0.234 (0.224, 0.244) | 0.134 (0.127, 0.142) |
| 2006 | 0.011 (0.009, 0.013) | 0.057 (0.053, 0.06) | 0.151 (0.145, 0.156) | 0.236 (0.228, 0.243) | 0.299 (0.289, 0.308) | 0.244 (0.234, 0.255) | 0.149 (0.141, 0.157) |
| 2007 | 0.012 (0.01, 0.015) | 0.057 (0.054, 0.061) | 0.157 (0.151, 0.162) | 0.243 (0.235, 0.251) | 0.313 (0.303, 0.322) | 0.264 (0.254, 0.275) | 0.161 (0.153, 0.169) |
| 2008 | 0.011 (0.009, 0.014) | 0.058 (0.055, 0.062) | 0.164 (0.158, 0.17) | 0.255 (0.247, 0.263) | 0.329 (0.319, 0.339) | 0.286 (0.275, 0.297) | 0.171 (0.163, 0.18) |
| 2009 | 0.013 (0.011, 0.015) | 0.062 (0.058, 0.065) | 0.171 (0.165, 0.177) | 0.265 (0.257, 0.273) | 0.346 (0.336, 0.356) | 0.308 (0.297, 0.319) | 0.182 (0.173, 0.191) |
| 2010 | 0.013 (0.011, 0.015) | 0.064 (0.061, 0.068) | 0.177 (0.171, 0.183) | 0.275 (0.267, 0.283) | 0.364 (0.354, 0.374) | 0.335 (0.323, 0.346) | 0.191 (0.182, 0.2) |
| 2011 | 0.013 (0.011, 0.015) | 0.066 (0.062, 0.07) | 0.183 (0.177, 0.19) | 0.287 (0.279, 0.295) | 0.38 (0.37, 0.391) | 0.358 (0.346, 0.369) | 0.207 (0.198, 0.216) |
| 2012 | 0.012 (0.01, 0.014) | 0.069 (0.066, 0.073) | 0.187 (0.181, 0.194) | 0.295 (0.287, 0.303) | 0.394 (0.383, 0.404) | 0.382 (0.371, 0.394) | 0.22 (0.21, 0.229) |
| 2013 | 0.013 (0.011, 0.015) | 0.075 (0.071, 0.079) | 0.198 (0.191, 0.205) | 0.315 (0.307, 0.323) | 0.42 (0.409, 0.431) | 0.407 (0.395, 0.419) | 0.249 (0.239, 0.26) |
| 2014 | 0.013 (0.011, 0.016) | 0.077 (0.073, 0.081) | 0.203 (0.196, 0.21) | 0.331 (0.323, 0.34) | 0.429 (0.418, 0.44) | 0.433 (0.42, 0.445) | 0.265 (0.255, 0.275) |
| 2015 | 0.015 (0.013, 0.018) | 0.079 (0.075, 0.083) | 0.207 (0.2, 0.213) | 0.347 (0.339, 0.356) | 0.443 (0.432, 0.454) | 0.452 (0.439, 0.464) | 0.286 (0.275, 0.297) |
| 2016 | 0.014 (0.012, 0.016) | 0.084 (0.08, 0.088) | 0.203 (0.196, 0.209) | 0.364 (0.356, 0.373) | 0.456 (0.445, 0.467) | 0.471 (0.458, 0.483) | 0.305 (0.294, 0.316) |
| 2017 | 0.015 (0.013, 0.017) | 0.087 (0.083, 0.091) | 0.206 (0.199, 0.213) | 0.371 (0.363, 0.38) | 0.472 (0.461, 0.483) | 0.489 (0.477, 0.502) | 0.326 (0.315, 0.338) |
| 2018 | 0.017 (0.015, 0.02) | 0.088 (0.084, 0.093) | 0.211 (0.204, 0.217) | 0.385 (0.376, 0.394) | 0.487 (0.476, 0.498) | 0.509 (0.496, 0.522) | 0.347 (0.336, 0.359) |
| 2019 | 0.017 (0.015, 0.019) | 0.088 (0.084, 0.092) | 0.216 (0.21, 0.223) | 0.39 (0.381, 0.399) | 0.5 (0.489, 0.511) | 0.528 (0.515, 0.541) | 0.368 (0.356, 0.38) |
| 2020 | 0.015 (0.013, 0.017) | 0.081 (0.077, 0.085) | 0.209 (0.203, 0.216) | 0.379 (0.37, 0.388) | 0.498 (0.487, 0.509) | 0.529 (0.516, 0.542) | 0.381 (0.369, 0.393) |

Data are percentages (95% confidence intervals).

**Supplementary Table 23. Point Prevalence of Axial Spondyloarthritis Diagnoses, Overall and Stratified by Sex**

| **Year** | **Overall Crude** | **Overall Age-Sex Standardised** | **Male** | **Female** |
| --- | --- | --- | --- | --- |
| 2004 | 0.081 (0.079, 0.082) | 0.081 (0.079, 0.083) | 0.128 (0.124, 0.131) | 0.034 (0.032, 0.036) |
| 2005 | 0.082 (0.08, 0.084) | 0.083 (0.081, 0.085) | 0.13 (0.127, 0.133) | 0.035 (0.034, 0.037) |
| 2006 | 0.085 (0.083, 0.087) | 0.086 (0.084, 0.088) | 0.134 (0.13, 0.137) | 0.036 (0.035, 0.038) |
| 2007 | 0.087 (0.085, 0.088) | 0.088 (0.086, 0.09) | 0.136 (0.133, 0.139) | 0.038 (0.036, 0.04) |
| 2008 | 0.089 (0.087, 0.091) | 0.091 (0.089, 0.092) | 0.139 (0.136, 0.143) | 0.039 (0.037, 0.041) |
| 2009 | 0.09 (0.088, 0.092) | 0.093 (0.091, 0.095) | 0.141 (0.138, 0.145) | 0.04 (0.038, 0.042) |
| 2010 | 0.091 (0.089, 0.093) | 0.094 (0.092, 0.096) | 0.143 (0.139, 0.146) | 0.041 (0.039, 0.043) |
| 2011 | 0.093 (0.092, 0.095) | 0.096 (0.094, 0.098) | 0.145 (0.142, 0.149) | 0.043 (0.041, 0.044) |
| 2012 | 0.095 (0.093, 0.097) | 0.098 (0.096, 0.1) | 0.147 (0.144, 0.151) | 0.044 (0.042, 0.046) |
| 2013 | 0.099 (0.097, 0.101) | 0.102 (0.1, 0.104) | 0.153 (0.149, 0.156) | 0.047 (0.045, 0.049) |
| 2014 | 0.102 (0.1, 0.104) | 0.104 (0.102, 0.106) | 0.156 (0.152, 0.159) | 0.049 (0.047, 0.051) |
| 2015 | 0.105 (0.103, 0.107) | 0.107 (0.105, 0.109) | 0.158 (0.155, 0.162) | 0.052 (0.05, 0.054) |
| 2016 | 0.107 (0.105, 0.109) | 0.109 (0.107, 0.111) | 0.16 (0.157, 0.164) | 0.055 (0.053, 0.057) |
| 2017 | 0.11 (0.108, 0.112) | 0.112 (0.11, 0.114) | 0.162 (0.159, 0.165) | 0.058 (0.056, 0.061) |
| 2018 | 0.112 (0.11, 0.114) | 0.114 (0.112, 0.116) | 0.163 (0.16, 0.166) | 0.062 (0.06, 0.064) |
| 2019 | 0.112 (0.11, 0.114) | 0.115 (0.113, 0.117) | 0.162 (0.158, 0.165) | 0.063 (0.061, 0.066) |
| 2020 | 0.113 (0.111, 0.115) | 0.115 (0.113, 0.117) | 0.16 (0.157, 0.164) | 0.065 (0.063, 0.067) |

Data are percentages (95% confidence intervals).

**Supplementary Table 24. Point Prevalence of Axial Spondyloarthritis Diagnoses Stratified by Age Categories**

| **Year** | **<25** | **25-35** | **35-45** | **45-55** | **55-65** | **65-75** | **75+** |
| --- | --- | --- | --- | --- | --- | --- | --- |
| 2004 | 0.011 (0.009, 0.013) | 0.044 (0.041, 0.047) | 0.089 (0.084, 0.093) | 0.131 (0.125, 0.137) | 0.14 (0.133, 0.147) | 0.09 (0.084, 0.096) | 0.036 (0.032, 0.041) |
| 2005 | 0.009 (0.007, 0.011) | 0.044 (0.041, 0.048) | 0.09 (0.085, 0.094) | 0.132 (0.126, 0.138) | 0.145 (0.138, 0.152) | 0.094 (0.088, 0.101) | 0.04 (0.036, 0.045) |
| 2006 | 0.009 (0.008, 0.011) | 0.044 (0.041, 0.048) | 0.092 (0.087, 0.096) | 0.129 (0.124, 0.135) | 0.152 (0.145, 0.159) | 0.106 (0.1, 0.113) | 0.041 (0.037, 0.046) |
| 2007 | 0.008 (0.007, 0.01) | 0.045 (0.042, 0.049) | 0.093 (0.088, 0.097) | 0.128 (0.123, 0.134) | 0.157 (0.15, 0.164) | 0.113 (0.106, 0.12) | 0.045 (0.041, 0.05) |
| 2008 | 0.009 (0.007, 0.011) | 0.045 (0.042, 0.048) | 0.094 (0.089, 0.098) | 0.128 (0.123, 0.134) | 0.161 (0.155, 0.168) | 0.121 (0.114, 0.129) | 0.05 (0.045, 0.055) |
| 2009 | 0.009 (0.007, 0.011) | 0.047 (0.044, 0.05) | 0.093 (0.088, 0.097) | 0.13 (0.124, 0.135) | 0.164 (0.157, 0.171) | 0.128 (0.121, 0.135) | 0.054 (0.05, 0.059) |
| 2010 | 0.008 (0.006, 0.009) | 0.049 (0.046, 0.052) | 0.092 (0.088, 0.097) | 0.128 (0.123, 0.134) | 0.163 (0.157, 0.17) | 0.133 (0.126, 0.141) | 0.059 (0.054, 0.064) |
| 2011 | 0.009 (0.007, 0.01) | 0.049 (0.045, 0.052) | 0.094 (0.09, 0.099) | 0.132 (0.126, 0.137) | 0.161 (0.154, 0.168) | 0.143 (0.135, 0.15) | 0.064 (0.059, 0.069) |
| 2012 | 0.009 (0.007, 0.01) | 0.051 (0.048, 0.054) | 0.094 (0.089, 0.098) | 0.133 (0.128, 0.139) | 0.16 (0.153, 0.167) | 0.152 (0.145, 0.16) | 0.067 (0.062, 0.073) |
| 2013 | 0.01 (0.008, 0.012) | 0.051 (0.048, 0.055) | 0.099 (0.095, 0.104) | 0.134 (0.129, 0.14) | 0.166 (0.159, 0.173) | 0.158 (0.151, 0.166) | 0.076 (0.07, 0.081) |
| 2014 | 0.012 (0.01, 0.014) | 0.053 (0.05, 0.057) | 0.102 (0.097, 0.107) | 0.136 (0.13, 0.141) | 0.168 (0.161, 0.175) | 0.162 (0.155, 0.17) | 0.079 (0.073, 0.084) |
| 2015 | 0.012 (0.01, 0.014) | 0.056 (0.053, 0.06) | 0.107 (0.102, 0.112) | 0.138 (0.133, 0.143) | 0.168 (0.161, 0.175) | 0.167 (0.159, 0.174) | 0.086 (0.08, 0.092) |
| 2016 | 0.013 (0.011, 0.015) | 0.058 (0.055, 0.061) | 0.11 (0.105, 0.115) | 0.14 (0.135, 0.146) | 0.165 (0.159, 0.172) | 0.171 (0.164, 0.179) | 0.092 (0.086, 0.099) |
| 2017 | 0.012 (0.01, 0.014) | 0.06 (0.057, 0.064) | 0.112 (0.108, 0.117) | 0.146 (0.14, 0.151) | 0.163 (0.157, 0.17) | 0.179 (0.172, 0.187) | 0.097 (0.091, 0.103) |
| 2018 | 0.012 (0.01, 0.014) | 0.061 (0.057, 0.064) | 0.116 (0.111, 0.121) | 0.147 (0.141, 0.152) | 0.166 (0.16, 0.173) | 0.182 (0.175, 0.19) | 0.103 (0.097, 0.11) |
| 2019 | 0.012 (0.01, 0.014) | 0.059 (0.056, 0.063) | 0.117 (0.112, 0.122) | 0.15 (0.145, 0.156) | 0.165 (0.159, 0.171) | 0.183 (0.176, 0.191) | 0.106 (0.1, 0.113) |
| 2020 | 0.011 (0.009, 0.013) | 0.055 (0.052, 0.059) | 0.116 (0.111, 0.121) | 0.151 (0.145, 0.157) | 0.165 (0.159, 0.172) | 0.183 (0.176, 0.191) | 0.113 (0.107, 0.12) |

Data are percentages (95% confidence intervals).

**Supplementary Table 25. Frequency Counts for the Main Three Initial Read/SNOMED Inflammatory Arthritis Codes Used in Incident Diagnoses in RA, PsA, and Axial SpA in Each Calendar Year**

| **SNOMED Concept ID** | **SNOMED Description ID** | **Term** | **Year** | | | | | | | | | | | | | | | | |
| --- | --- | --- | --- | --- | --- | --- | --- | --- | --- | --- | --- | --- | --- | --- | --- | --- | --- | --- | --- |
|  |  |  | **2004** | **2005** | **2006** | **2007** | **2008** | **2009** | **2010** | **2011** | **2012** | **2013** | **2014** | **2015** | **2016** | **2017** | **2018** | **2019** | **2020** |
| **Rheumatoid Arthritis** | | | | | | | | | | | | | | | | | | | |
| 69896004 | 116082011 | rheumatoid arthritis | 5571 (78.83) | 5143 (78.7) | 4708 (78.74) | 4486 (76.84) | 4524 (77.07) | 4698 (76.07) | 4562 (74.73) | 4346 (71.25) | 4251 (67.14) | 4260 (40.07) | 4062 (42.05) | 3987 (47.13) | 3678 (47.2) | 3532 (46.92) | 3410 (33.95) | 3236 (39.8) | 2520 (52.68) |
| 239791005 | 359291017 | seropositive rheumatoid arthritis, unspecified | 256 (3.62) | 223 (3.41) | 214 (3.58) | 247 (4.23) | 237 (4.04) | 271 (4.39) | 274 (4.49) | 299 (4.9) | 291 (4.6) | 399 (3.75) | 363 (3.76) | 413 (4.88) | 396 (5.08) | 452 (6) | 490 (4.88) | 495 (6.09) | 471 (9.85) |
| 239792003 | 359292012 | seronegative rheumatoid arthritis | 453 (6.41) | 460 (7.04) | 413 (6.91) | 480 (8.22) | 523 (8.91) | 633 (10.25) | 607 (9.94) | 679 (11.13) | 817 (12.9) | 819 (7.7) | 841 (8.71) | 919 (10.86) | 994 (12.76) | 926 (12.3) | 970 (9.66) | 878 (10.8) | 485 (10.14) |
| **Psoriatic Arthritis** | | | | | | | | | | | | | | | | | | | |
| 33339001 | 55626019 | psoriasis with arthropathy | 15 (0.79) | 19 (1.07) | 15 (0.9) | 16 (0.9) | 14 (0.74) | 7 (0.34) | 14 (0.68) | 19 (0.89) | 18 (0.91) | 10 (0.45) | 16 (0.74) | 27 (1.18) | 25 (1.15) | 22 (1.01) | 24 (1.1) | 49 (2.38) | 471 (71.15) |
| 33339001 | 55627011 | psoriatic arthropathy | 1633 (86.45) | 1519 (85.48) | 1411 (84.34) | 1462 (82.09) | 1570 (82.89) | 1251 (61.02) | 1115 (54.13) | 1165 (54.31) | 1054 (53.02) | 1110 (50.09) | 1165 (54.21) | 1229 (53.76) | 1091 (50.18) | 1045 (48.16) | 974 (44.66) | 825 (40.15) | 93 (14.05) |
| 33339001 | 55628018 | psoriatic arthritis | 135 (7.15) | 137 (7.71) | 164 (9.8) | 208 (11.68) | 216 (11.4) | 721 (35.17) | 860 (41.75) | 908 (42.33) | 860 (43.26) | 1005 (45.35) | 899 (41.83) | 963 (42.13) | 969 (44.57) | 1018 (46.91) | 1104 (50.62) | 1112 (54.11) | 90 (13.6) |
| **Axial Spondyloarthritis** | | | | | | | | | | | | | | | | | | | |
| 9631008 | 16833013 | ankylosing spondylitis | 977 (99.39) | 952 (99.37) | 977 (99.39) | 896 (99.67) | 978 (99.39) | 931 (98.62) | 92 (99.33) | 917 (98.6) | 935 (98.52) | 944 (98.23) | 1041 (93.2) | 1050 (85.37) | 962 (79.83) | 934 (77.06) | 920 (70.93) | 888 (69.38) | 636 (65.63) |
| 713777005 | 3297625017 | non-radiographic axial spondyloarthritis | <5 | <5 | <5 | <5 | <5 | <5 | <5 | <5 | <5 | <5 | <5 | 6 (0.49) | 9 (0.75) | 13 (1.07) | 17 (1.31) | 17 (1.33) | 28 (2.89) |
| 723116002 | 3334645019 | axial spondyloarthritis | 5 (0.51) | 6 (0.63) | 5 (0.51) | <5 | 6 (0.61) | 12 (1.27) | <5 | 12 (1.29) | 13 (1.37) | 17 (1.77) | 74 (6.62) | 174 (14.15) | 233 (19.34) | 265 (21.86) | 360 (27.76) | 375 (29.3) | 305 (31.48) |

**Supplementary Figure 1. Annual Incidence of Rheumatoid Arthritis, Psoriatic Arthritis, and Axial Spondyloarthritis Diagnoses in Males and Females Stratified by Age Groups.**

**
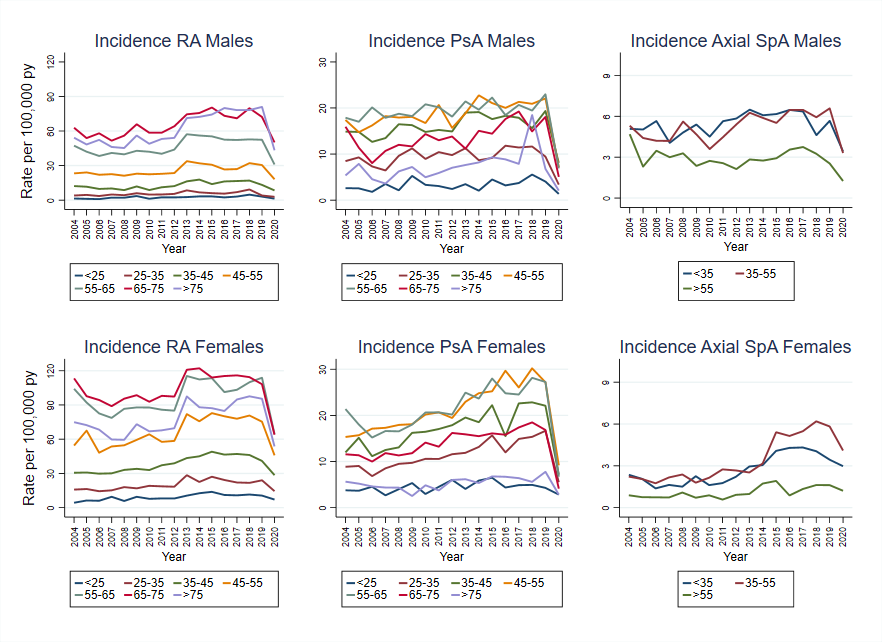
**

For RA and PsA - 25-35: ≥25 to <35 years; 35-45: ≥35 to <45 years; 45-55: ≥45 to <55 years; 55-65: ≥55 to <65 years; 65-75: ≥65 to <75 years; >75: ≥75 years; for axial SpA - 35-55: ≥35 to<55 years; >55: ≥55 years; py = person-years.

**Supplementary Figure 2. Point Prevalence of Rheumatoid Arthritis, Psoriatic Arthritis, and Axial Spondyloarthritis Diagnoses in Males and Females Stratified by Age Groups.**

**
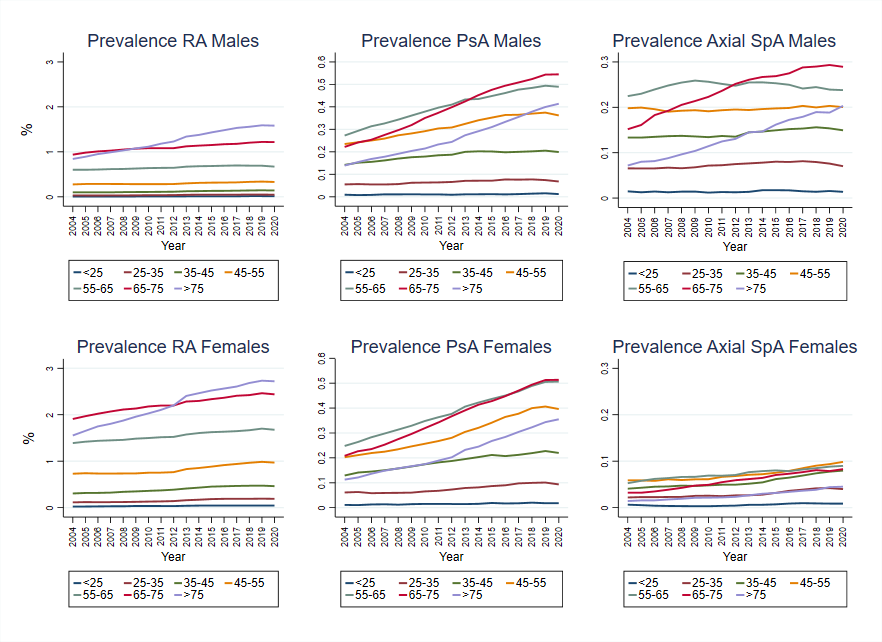
**

25-35: ≥25 to <35 years; 35-45: ≥35 to <45 years; 45-55: ≥45 to <55 years; 55-65: ≥55 to <65 years; 65-75: ≥65 to <75 years; >75: ≥75 years.

**REFERENCES**

1. NHS Digital. SNOMED CT [Internet]. Available at https://digital.nhs.uk/services/terminology-and-classifications/snomed-ct [accessed 2022 Apr 10].

2. Muller S. An algorithm to identify rheumatoid arthritis in primary care: a Clinical Practice Research Datalink study. BMJ Open. 2015 Dec 1;5(12):e009309.

3. Ogdie A. Validity of psoriatic arthritis and capture of disease modifying antirheumatic drugs in the health improvement network. Pharmacoepidemiol Drug Saf. 2014 Sep 1;23(9):918–22.
